# Receptor-interacting protein kinase 1 is a key mediator in TLR3 ligand and Smac mimetic-induced cell death and suppresses TLR3 ligand-promoted invasion in cholangiocarcinoma

**DOI:** 10.1186/s12964-020-00661-3

**Published:** 2020-10-09

**Authors:** Thanpisit Lomphithak, Swati Choksi, Apiwat Mutirangura, Rutaiwan Tohtong, Tewin Tencomnao, Hajime Usubuchi, Michiaki Unno, Hironobu Sasano, Siriporn Jitkaew

**Affiliations:** 1 Graduate Program in Clinical Biochemistry and Molecular Medicine, Department of Clinical Chemistry, Faculty of Allied Health Sciences, Chulalongkorn University, Bangkok, 10330 Thailand; 2 Laboratory of Immune Cell Biology, Center for Cancer Research, National Cancer Institute, National Institutes of Health, 37 Convent Drive, Bethesda, MD 20892 USA; 3 Department of Anatomy, Faculty of Medicine, Center of Excellence in Molecular Genetics of Cancer and Human Diseases, Chulalongkorn University, Bangkok, 10330 Thailand; 4 Department of Biochemistry, Faculty of Science, Mahidol University, Bangkok, 10400 Thailand; 5 Age-Related Inflammation and Degeneration Research Unit, Department of Clinical Chemistry, Faculty of Allied Health Sciences, Chulalongkorn University, Bangkok, 10330 Thailand; 6 Department of Pathology, Tohoku University School of Medicine, Sendai, Miyagi 980-8575 Japan; 7 Department of Surgery, Tohoku University School of Medicine, Sendai, Miyagi 98-8075 Japan

**Keywords:** Toll-like receptor 3, Smac mimetic, Receptor-interacting protein kinase 1 (RIPK1), Necroptosis, Invasion, Cholangiocarcinoma

## Abstract

**Background:**

Toll-like receptor 3 (TLR3) ligand which activates TLR3 signaling induces both cancer cell death and activates anti-tumor immunity. However, TLR3 signaling can also harbor pro-tumorigenic consequences. Therefore, we examined the status of TLR3 in cholangiocarcinoma (CCA) cases to better understand TLR3 signaling and explore the potential therapeutic target in CCA.

**Methods:**

The expression of TLR3 and receptor-interacting protein kinase 1 (RIPK1) in primary CCA tissues was assayed by Immunohistochemical staining and their associations with clinicopathological characteristics and survival data were evaluated. The effects of TLR3 ligand, Poly(I:C) and Smac mimetic, an IAP antagonist on CCA cell death and invasion were determined by cell death detection methods and Transwell invasion assay, respectively. Both genetic and pharmacological inhibition of RIPK1, RIPK3 and MLKL and inhibitors targeting NF-κB and MAPK signaling were used to investigate the underlying mechanisms.

**Results:**

TLR3 was significantly higher expressed in tumor than adjacent normal tissues. We demonstrated in a panel of CCA cell lines that TLR3 was frequently expressed in CCA cell lines, but was not detected in a nontumor cholangiocyte. Subsequent in vitro study demonstrated that Poly(I:C) specifically induced CCA cell death, but only when cIAPs were removed by Smac mimetic. Cell death was also switched from apoptosis to necroptosis when caspases were inhibited in CCA cells-expressing RIPK3. In addition, RIPK1 was required for Poly(I:C) and Smac mimetic-induced apoptosis and necroptosis. Of particular interest, high TLR3 or low RIPK1 status in CCA patients was associated with more invasiveness. In vitro invasion demonstrated that Poly(I:C)-induced invasion through NF-κB and MAPK signaling. Furthermore, the loss of RIPK1 enhanced Poly(I:C)-induced invasion and ERK activation in vitro. Smac mimetic also reversed Poly(I:C)-induced invasion, partly mediated by RIPK1. Finally, a subgroup of patients with high TLR3 and high RIPK1 had a trend toward longer disease-free survival (*p* = 0.078, 28.0 months and 10.9 months).

**Conclusion:**

RIPK1 plays a pivotal role in TLR3 ligand, Poly(I:C)-induced cell death when cIAPs activity was inhibited and loss of RIPK1 enhanced Poly(I:C)-induced invasion which was partially reversed by Smac mimetic. Our results suggested that TLR3 ligand in combination with Smac mimetic could provide therapeutic benefits to the patients with CCA.

**Video abstract**

**Graphical abstract:**

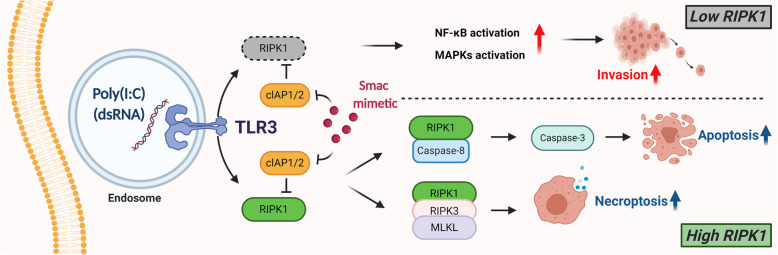

## Background

Cholangiocarcinoma (CCA), a markedly heterogeneous malignancy arising from the bile duct epithelium, occurs with a high incidence in Asian countries (incidence > 6 cases per 100,000 people) with the highest incidence in northeastern Thailand (incidence 85 cases per 100,000 people), but its overall incidence rate is increasing worldwide [1]. CCA has relatively high mortality with 5-year survival (7–20%) and recurrence/metastasis rates and subsequently poor prognosis [1]. The great majority of CCA patients are usually diagnosed at an advanced stage in which the first-line gemcitabine alone or combined gemcitabine and cisplatin chemotherapy, are not effective [2, 3] and therefore the better understanding of its molecular pathogenesis and subsequently the discovery of novel therapeutic targets are required to improve the clinical outcome of these patients [4, 5]. Chronic inflammation and immunosuppressive tumor microenvironment have been generally considered to play pivotal roles in the pathogenesis and/or development of CCA following primary sclerosing cholangitis, parasitic and viral infection, which also indicated the immune related etiology of CCA, and cancer immunotherapy was therefore proposed as an alternative strategy for the treatment of CCA patients [5–7]. In addition, development of a novel therapeutic approach, in particular the induction of immunogenic cell death (ICD) which could eliminate cancer cells and reactivate immune responses also result in improving the treatment efficacy, reduce recurrence and increase long-lasting survival rates of the patients [8].

Toll-like receptors (TLRs) have become interesting targets for cancer immunotherapy. Among TLRs, Toll-like receptor 3 (TLR3) is one of the promising targets that represents a potential for anti-tumor therapy. TLR3, an endosomal pattern recognition receptor, mediates both innate and adaptive immune responses by sensing viral double-stranded RNA (dsRNA), but also endogenous ligands found at site of damaged tissues and mRNA components released from dying cells [9, 10]. TLR3-mediated immune responses is also characterized by the production of inflammatory cytokines and type I interferons (IFNs) [11]. Upon activation, TLR3 signals through an adapter protein called TIR-domain-containing adapter-inducing interferon-β (TRIF also known TICAM 1) which then recruits receptor-interacting protein kinase 1 (RIPK1) and TNF receptor-associated factor 6 (TRAF6), thereby leading to the activation of nuclear factor kappaB (NF-κB), mitogen-activated protein kinase (MAPK), and interferon regulatory factor 3 (IRF3) inflammatory signaling pathways [12]. Therefore, TLR3 ligands have successfully been developed and approved at clinical setting as a synthetic dsRNA such as polyinosinic-polycytidylic acid, Poly(I:C), to mimic the response to RNA viral infection [13, 14]. In addition, TLR3 ligands have been studied in clinical trials as adjuvants for cancer immunotherapy to enhance cancer vaccine efficacy [15–17]. In addition to orchestrating inflammatory and immune responses, triggering TLR3 signaling by TLR3 ligands has been reported to directly kill various cancer cells such as breast cancer [18, 19], melanoma [20, 21], renal cell carcinoma (RCC) [22], prostate cancer [23, 24], nasopharyngeal carcinoma [25, 26], multiple myeloma [27], head and neck squamous cell carcinoma (HNSCC) [28–30], hepatocellular carcinoma (HCC) [31], neuroblastoma [32], non-small cell lung cancer (NSCLC) [33–35], and mesothelioma [36]. TLR3 ligands-mediated cell death is involved the formation of a signaling complex composed of TRIF, RIPK1, Fas-associated protein with death domain (FADD) and caspase-8, the death signaling complex also called ripoptosome [34, 37]. RIPK1 represents a key scaffold molecule linking TLR3/TRIF to FADD/caspase-8 signaling cascade which then triggers caspase-8-dependent extrinsic apoptosis [38]. When caspase activity is inhibited, RIPK1 can form a cytosolic death signaling complex with receptor-interacting protein kinase 3 (RIPK3) and mixed lineage kinase domain-like protein (MLKL) which then induces another mode of programmed cell death called necroptosis [39]. As a consequence, necroptotic cell death has been reported to enhance anti-tumor immunity, as in the basic concept of cancer vaccine immunotherapy and is therefore considered an immunogenic cell death (ICD) [40–44]. However, TLR3 ligands-mediated necroptosis has not been well explored in cancer cells [45, 46].

Negative regulators of TLR3 ligands-mediated apoptosis have been reported in the literature. Cellular inhibitor of apoptosis proteins (cIAPs) including cIAP1 and cIAP2 represent two key molecules that limit TLR3 ligands-mediated apoptosis. Both cIAP1 and cIAP2 harbor a really interesting new gene (RING) domain, an E3 ubiquitin ligase [47], thereby mediating RIPK1 poly-ubiquitination resulting in the negative regulation by preventing RIPK1 to form a cytosolic death complex as reported in TNFR1 signaling complex. Therefore, small molecule antagonists of IAPs also known as Smac mimetics have been developed to overcome apoptosis resistance. In TNFR1 signaling complex, Smac mimetics trigger the auto-ubiquitination and proteasomal degradation of E3 ligases cIAP1 and cIAP2 that promote RIPK1 de-ubiquitination, hence its releasing to a cytosolic death signaling complex [48]. Accordingly, Smac mimetics have been reported to sensitize TLR3 ligands-induced apoptosis in some cancer cells [21, 26, 33, 34, 37, 49]. Moreover, recent study has demonstrated that the removal of cellular FLICE-like inhibitory protein (c-FLIP), a strong negative regulator of caspase-8-mediated apoptosis could overcome the resistance to TLR3 ligands-mediated apoptosis [33, 36].

RIPK1, a serine/threonine kinase is a multifunctional protein that regulates signaling pathways leading to opposing outcomes including inflammation and cell death both in the form of apoptosis and necroptosis [50]. RIPK1 regulates signaling pathways through either kinase-dependent or kinase-independent manner. In addition to TNF-α signaling, RIPK1 has been reported to be also required for TLR3-mediated NF-κB activation [51] and cell death [34, 37, 52]. The roles of RIPK1 and its regulation are best characterized in TNF-α signaling but its roles in TLR3 signaling have been less studied. In addition, whether RIPK1 and its interplay with TLR3 could play a role in CCA and regulate cancer cell invasion have also remained largely unknown [53, 54].

TLR3 has been reported as one of novel therapeutic targets that can eliminate cancer cells and activate anti-tumor immunity [55]. Therefore, in this study, we first examined TLR3 status in CCA cases and evaluated its association with clinicopathological parameters of the individual patients in order to search for a novel therapeutic target and also gain a better understanding of TLR3 signaling for improvement of therapeutic approaches targeting TLR3 with TLR3 ligands. Therapeutic targeting TLR3 by the combination of TLR3 ligand, Poly(I:C) and an IAPs antagonist, Smac mimetic to induce cell death and modulate invasion were also investigated in CCA cells. In addition, RIPK1 status in CCA cases and its interplay with TLR3 in the modulation of tumorigenic properties, in particular invasion were also explored in this study.

## Methods

### Cell culture and treatment

Human CCA cell lines (KKU213, KKU100, KKU214, KKU-M055, HuCCT-1) and a nontumor human cholangiocyte cell line (MMNK1) were obtained from the Japanese Collection of Research Bioresources (JCRB) Cell Bank, Osaka, Japan. RMCCA-1 cells were developed from Thai patients with CCA [56]. All human CCA cell lines and MMNK1 were cultured in HAM’s F-12 medium (HyClone Laboratories, Logan, Utah, USA). All culture media were supplemented with 10% fetal bovine serum (Sigma, St Louis, Missouri, USA) and 1% Penicillin-Streptomycin (HyClone Laboratories, Logan, Utah, USA). All cells were cultured in a humidified incubator at 37 °C with 5% CO_2_. All cell lines were tested for mycoplasma contamination and were mycoplasma free. For drug treatment, cells were pretreated with Smac mimetic, SM-164 (5 nM for KKU213 or 50 nM for KKU100, HuCCT-1 and MMNK1) or Smac mimetic and zVAD-fmk (20 μM) for 2 h, after that cells were transfected with 2.5 μg/ml Poly(I:C) by TurboFect transfection reagent (Thermo fisher scientific, Waltham, Massachusetts, USA). Combination index (CI) for Poly(I:C) and Smac mimetic combination treatment was calculated based on Chou-Talalay method using CompuSyn version 1.0 software [57].

### Reagents and antibodies

Poly(I:C) HMW was purchased from InvivoGen (San Diego, California, USA). Smac mimetic (SM-164) was a gift from S. Wang (University of Michigan, Ann Arbor, Michigan, USA). Pan-caspase inhibitor (z-VAD-FMK), GSK’782, necrosulfonamide (NSA), Bay11–7082, U0126, SP600125 and SB203580 were purchased from Calbiochem (Merck Millipore, Darmstadt, Germany). Necrostatin-1 (Nec-1) were purchased from Sigma (St Louis, Missouri, USA). TNF-α was purchased from R&D systems (Minneapolis, Minnesota, USA). TLR3 inhibitor (CuCpt4a) was purchased from APExBIO (Boston, Massachusetts, USA). Antibodies for Western blot were purchased from commercial available providers as following: anti-RIPK1 (610459) was from BD Biosciences (San Jose, California, USA); anti-TLR3 (6961), anti-RIPK3 (8457), anti-cIAP1 (7065), anti-cIAP2 (3130), anti-caspase-8 (9746), anti-caspase-3 (9662), anti-PARP-1 (9542) and anti-actin (4970) were from Cell signaling (Danvers, Massachusetts, USA); anti-MLKL (ab184718) was from Abcam (Cambridge, UK).

### Patient samples

Formalin-fixed and paraffin-embedded tumor blocked were obtained from 88 CCA patients (Intrahepatic CCA = 21 samples and Hilar CCA = 67 samples) whose primary tumor were surgically resected between 2005 and 2015 at Tohoku University Hospital, Sendai, Japan. Clinicopathological parameters of individual patient were detailed in Table 1. The study protocol was approved by IRB of Tohoku University School of Medicine, Sendai, Japan. Informed consent was obtained.

**Table 1:** Associations of RIPK1 and TLR3 expression with clinicopathological parameters of CCA patients

		TLR3		Chi square	*p-*value	RIPK1		Chi square	*p-*value
**Gender**	n (%)	Low	High			Low	High		
Male	54 (39%)	22	30	0.103	0.748	26	26	1.059	0.303
Female	35 (61%)	14	22			22	14		
**Age (years)**									
< 67	44 (50%)	19	25	0.188	0.665	28	16	2.933	0.087
> = 67	44 (50%)	17	27			20	24		
**Grading**									
well differentiated	13 (14.8%)	4	11	3.152	0.207	9	6	0.277	0.893
moderately differentiated	73 (83%)	32	39			38	33		
poorly differentiated	2 (2.2%)	0	2			1	1		
**Tumor size (mm)**									
< 35	30 (38.5%)	14	35	5.727	**0.030**	27	22	0.119	0.730
> = 35	48 (61.5%)	20	17			19	18		
**Perineural Invasion**									
Present	65 (73%)	22	43	5.132	**0.023**	43	22	13.517	**0.0002**
None	24 (27%)	14	9			5	18		
**Vascular Invasion**									
Present	66 (74.2%)	21	45	9.026	**0.003**	41	25	6.111	**0.013**
None	23 (25.8%)	15	7			7	15		
**Lymph node invasion**									
Present	62 (69.7%)	19	43	9.145	**0.002**	40	22	8.414	**0.004**
None	27 (30.3%)	17	9			8	18		

### Immunohistochemical staining and evaluation

Paraffin-embedded CCA sections were deparaffinized and hydrated in xylene and ethanol respectively, then autoclaved for 5 min in an antigen retrieval solution, sodium citrate buffer (pH 6.0). Tissue sections were incubated overnight at 4 °C with primary antibodies, including mouse monoclonal anti-TLR3 (1:500 dilution; ab13915; Abcam, Cambridge, UK) and mouse anti-RIP (1:200 dilution; 610459; BD Biosciences, San Jose, California, USA). Tissue sections were then incubated with biotinylated secondary antibody. After that, peroxidase activity was developed with 3,30-diaminobenzidine tetrahydrochloride and counterstained with hematoxylin. Tissue sections were then sealed with neutral resins.

Stained slides were evaluated by light microscopy by two individuals (HU and TL). All tissue sections were scored in a semi-quantitative manner. Intensity was classified as 0 (no-stain), + 1 (weak staining), + 2 (moderate staining), or + 3 (strong staining). A value H-score was obtained for each slide by using the following formula: H-score = (%Strong × 3) + (%Moderate × 2) + %Weak. Low and high TLR3 or RIPK1 expression were divided based on the median H-score of all specimens.

### CRISPR plasmid, shRNAs and Lentivirus infection

CRISPR plasmids targeting human RIPK1 (NM_003804) and human RIPK3 (NM_006871) were generated following Zhang’s protocol [58]. The sequence for CRISPR-RIPK1 was 5′-CACC GGATGCACGTGCTGAAAGCCG-3′ and CRISPR-RIPK3 was 5′-CAGTGTTCCGGGCGCA AAT-3′. The shRNAs against human MLKL (NM_152649.4) corresponding to the 3′ untranslated region 2025–2045 (shMLKL1) and 1907–1927 (shMLKL2) were purchased from Sigma (St Louis, Missouri, USA). All plasmid constructs were subsequently confirmed by DNA sequencing. HEK293T cells were used to generate lentiviral particles, by co-transfection of packaging plasmid (pCMV-VSV-G) and envelope plasmid (pCMV-dr8.2-dvpr) and either CRISPR-V2 or CRISPR-RIPK1 or CRISPR-RIPK3 plasmids or shRNA-non-targeting (shNT; pLKO.1puro) and shRNA-MLKL (shMLKL). After 24 h, supernatants containing viral particles were collected and filtered through a 0.45 μM sterile filter membrane (Merck Millipore, Darmstadt, Germany). Eight μg/ml of polybrene (Merck Millipore, Darmstadt, Germany) was added to the lentiviral preparation and then used to infect the cells. After 24 h of infection, the cells were selected with puromycin (Merck Millipore, Darmstadt, Germany) for a further 48 h.

### Western blot analysis

Western blot analysis was performed according to previously described [59]. Cells were lysed in RIPA buffer (Merck Millipore, Darmstadt, Germany) containing a proteinase inhibitor cocktail (Roche, Mannheim, Germany). Total proteins were separated by 10% or 12% SDS-PAGE and proteins were transferred onto PVDF membranes. The membranes were incubated with the primary antibodies listed above. The proteins were visualized by enhanced chemiluminescence according to the manufacturer’s instructions (Bio-Rad, Hercules, California, USA). All Western blots shown were representative of at least three independent experiments.

### AnnexinV and Propidium iodide (PI) staining

Cell death was assessed by AnnexinV and PI dual staining. Briefly, cells were collected and resuspended in Annexin V binding buffer containing recombinant Annexin V-FITC (ImmunoTools, Friesoythe, Germany) and PI (Invitrogen; Thermo Fisher Scientific, Inc., California, USA). The stained cells were analyzed with flow cytometry (Navios, Beckman Coulter, Indianapolis, USA). The percentage of cells with AnnexinV positive/PI negative and AnnexinV positive/PI positive were considered as cell death.

### Transwell invasion assay

Transwell insert was pre-coated with 50 μg/well of Matrigel (Corning, Tewksbury, Massachusetts, USA). Cells in serum free media were seeded in the upper Transwell chambers (Corning, Tewksbury, Massachusetts, USA). Complete medium was added to the lower chamber. The plates were incubated at 37 °C, 5% CO_2_. After 12 h, the invaded cells were fixed with 4% formaldehyde and stained with 0.1% crystal violet. Number of invaded cells were counted in 5 random fields.

### Statistical analysis

All statistical analyses were conducted using the software package SPSS for Windows. The Pearson’s χ2 was used to analyze the association of clinicopathological factors and TLR3 or RIPK1 expression. Disease free and overall survival of patients were estimated by Kaplan-Meier method using log-rank test. Results were expressed as the mean ± standard deviation (S.D.) of at least three independent experiments. Comparisons between two groups were determined by a two-tailed Student’s t-tests. All *p*-values less than 0.05 were considered statistically significant.

## Results

### TLR3 is frequently expressed in primary CCA tissues and cell lines

**Fig. 1:**
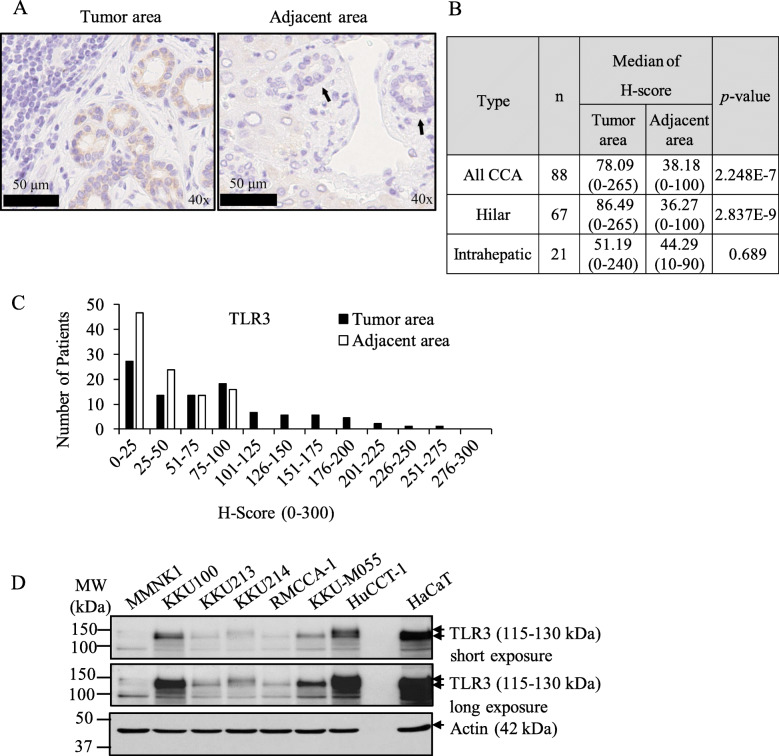
TLR3 is frequently expressed in primary CCA tissues and cell lines. **a** The representative cytoplasmic TLR3 immunostaining in tumor area and adjacent normal tissues. Black arrowheads indicate bile ducts. **b** The median of H-score of staining in CCA primary tissues (tumor tissues and adjacent). The difference between the median of H-score of tumor and adjacent tissues was calculated by one-way ANOVA and presented as *p*-value. **c** Distribution of TLR3 expression according to H-score of tumor area and adjacent area. **d** Protein expression of TLR3 was analyzed in 6 different human CCA cell lines and an immortalized human cholangiocyte cell line, MMNK1 using Western blot analysis and β-actin was served as loading control

Stimulation of TLR3 in cancer cells directly induced apoptosis and TLR3 expression in breast cancer patients has been reported to predict clinical responses to TLR3 ligand stimulation [18]. We therefore immunolocalized TLR3 in 88 CCA patients in this study. TLR3 was previously reported to be localized in both the endosomal compartments and on the cell surface. We demonstrated that TLR3 was predominantly immunolocalized in the cytoplasm of human CCA primary tissues (Fig. 1a and Fig. S1). The TLR3 immunoreactivity (combined hilar and intrahepatic CCA) was differentially detected in CCA cases (median of H-score of 78.09), while weakly present in adjacent tissues (median of H-score of 38.18) (Fig. 1b). TLR3 was significantly higher expressed in tumor tissues than adjacent tissues (*p* = 2.248E-7) (Fig. 1b). TLR3 intensity was differentially distributed from negative to strong intensity, compared with adjacent tissues demonstrating mostly negative and low intensity (Fig. 1c). We then evaluated the TLR3 expression in a panel of CCA cell lines and immortalized nontumor cholangiocytes by Western blot analysis. TLR3 was also differentially expressed in all CCA cell lines but not in nontumor cholangiocytes (Fig. 1d). Collectively, results of our present study demonstrated that TLR3 was differentially expressed in the great majority of CCA patients and all CCA cell lines examined but was restricted to nontumor cholangiocytes. Therefore, targeting TLR3 signaling could be a novel potential therapeutic target for CCA patients.

### TLR3 ligand, Poly(I:C) and Smac mimetic induce caspase-8 activation and apoptosis in CCA cell lines

**Fig. 2:**
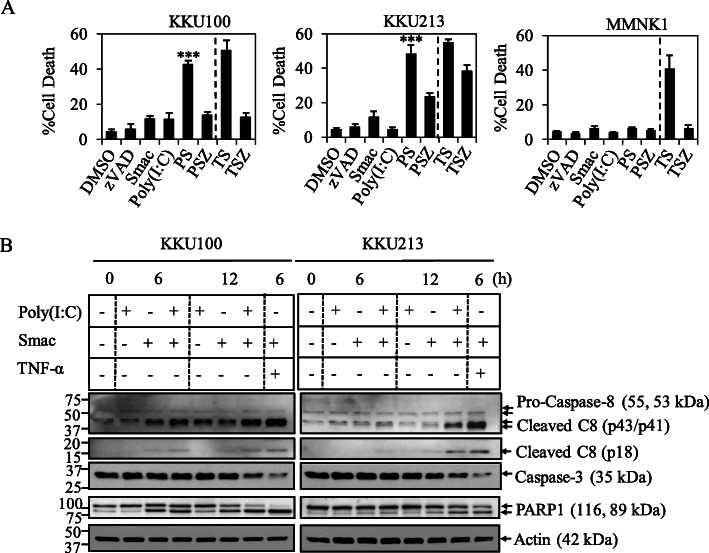
Smac mimetic sensitizes CCA cell lines to TLR3 ligand, Poly(I:C)-induced caspase-8 activation and apoptosis. **a** MMNK1, KKU100, and KKU213 cells were pretreated with Smac mimetic (50 nM MMNK1 and KKU100, and 5 nM KKU213) (Smac) or Smac mimetic and 20 μM zVAD-fmk (SZ) for 2 h. After that the cells were transfected with 2.5 μg/ml Poly(I:C) (PS, PSZ) for 24 h. TNF-α at 10 ng/ml and Smac mimetic or zVAD-fmk at the same concentration as with Poly(I:C) (TS, TSZ) were used as a positive control. Cell death was determined by Annexin V and propidium iodide staining followed by flow cytometry. Data from three independent experiments was presented as mean ± S.D.; * *p* < 0.05, **p < 0.01, *** *p* < 0.001 **b** KKU100 and KKU213 cells were treated as in (**a**) for 6 h and 12 h. Cell lysates were collected, after that the activation of caspase-8 and caspase-3 and cleavage of PARP-1 were analyzed by Western blot analysis. β-actin was served as loading control. Data shown was a representative of two independent experiments

In order to explore the sensitivity of CCA cells to TLR3 ligand, Poly(I:C), a commonly used ligand to activate TLR3, we found that none of CCA cell lines that were differentially expressed TLR3 were sensitive to Poly(I:C)-induced cell death both by direct treatment or transfection (Fig. S2A, B). We therefore tentatively hypothesized that the responsiveness to Poly(I:C) stimulation could be possibly influenced by negative regulators including cellular inhibitor of apoptosis proteins (cIAP1 and cIAP2) [37, 60] and cellular FLICE-like inhibitory protein (c-FLIP) [33, 36] (Fig. S3A, B). Therefore, a Smac mimetic (SM-164), an IAP antagonist was combined with Poly(I:C) to enhance the sensitivity of Poly(I:C)-induced cell death. Two of six CCA cell lines examined in this study including KKU100 and KKU213 were the most sensitive to the combination treatment as evaluated by a cell viability, MTT assay followed by KKU214 and RMCCA-1, while KKU-M055 and HuCCT-1 and a nontumor cholangiocyte, MMNK1 were less sensitive (Fig. S4A, B). We therefore selected KKU100 and KKU213 as two of representative CCA cell lines for the further study, whereas MMNK1 was included as a nontumor cholangiocyte control. We observed a stronger induction of cell death when Poly(I:C) was transfected with a lower dose at 2.5 μg/ml than that of a direct addition into culture media with a higher dose at 25 μg/ml, therefore transfection of Poly(I:C) was used in the rest of experiments which is consistent with a previous study in prostate cancer cells [61]. The combination index (CI index) was calculated to indicate the synergistic effects of the combination treatment [57] and the concentration of both Poly(I:C) and Smac mimetic yielding the highest synergistic effect was selected for further experiments (Fig. S5A, B, C). In order to further investigate whether Poly(I:C) and Smac mimetic specifically triggered apoptosis, more specific apoptosis assays were used to confirm an induction of apoptosis. As in cell viability assay, Poly(I:C) single treatment did not induce cell death, whereas Smac mimetic marginally induced cell death in both KKU100 and KKU213 as evaluated by Annexin V/PI staining (Fig. 2a). However, when Poly(I:C) was combined with Smac mimetic, the cell death was enormously increased in both KKU100 and KKU213, whereas MMNK1 remained completely resistant to Poly(I:C) and Smac mimetic treatment (Fig. 2a). On the contrary, TNF-α and Smac mimetic, a well-known inducer of apoptosis serving as a positive control also enormously induced cell death in MMNK1. In addition, Smac mimetic induced the degradation of cIAP1 and cIAP2 in all the cell lines examined in this study (Fig. S6A, B, C, D), while Poly(I:C) only triggered the upregulation of TLR3 in CCA cell lines, but not in MMNK1 (Fig. S7A, B, C, D). The pan-caspase inhibitor, zVAD-fmk completely protected cell death in KKU100, while partially inhibited cell death in KKU213 (Fig. 2a). Consistent with Annexin V/PI staining, activation of caspase-8 (p43/p41 and p18 fragments) and decreased pro-caspase-3 were both detected by Western blot at 6 h and 12 h after the addition of Poly(I:C) and Smac mimetic in both cell lines, and coincided with the cleavage of PARP-1, all characteristic features of apoptosis (Fig. 2b, Fig. S8A, B). Altogether, this set of experiments suggested that Poly(I:C) and Smac mimetic combination treatment triggers caspase-8 activation and apoptosis in CCA cell lines.

### TLR3 ligand, Poly(I:C) and Smac mimetic trigger necroptosis upon caspase inhibition in CCA cell lines

**Fig. 3:**
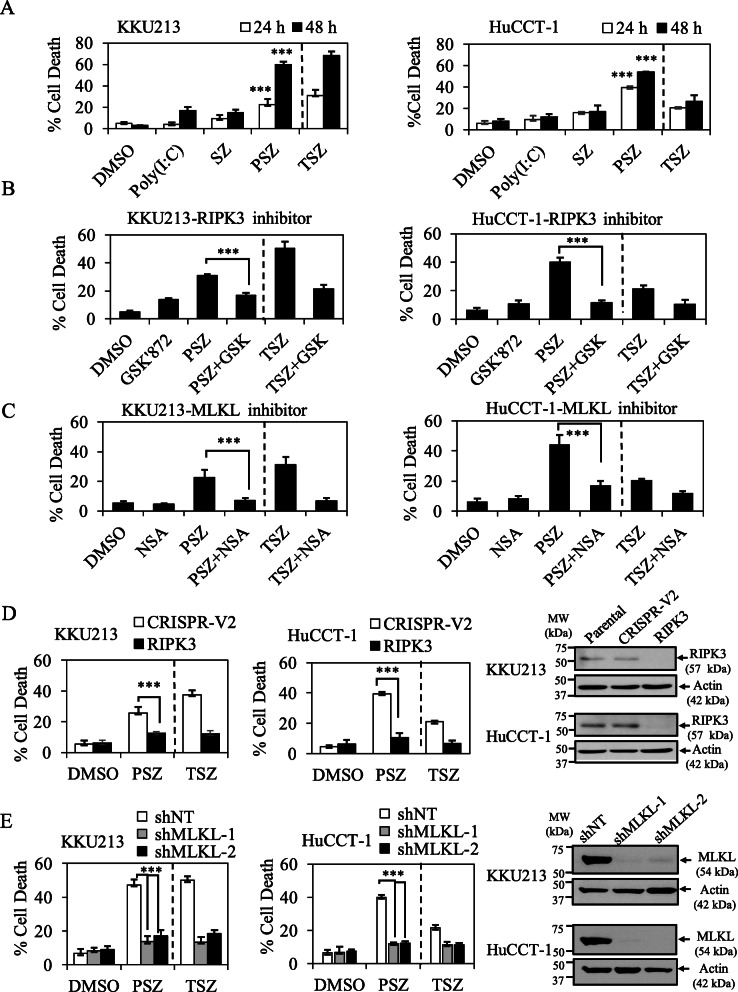
TLR3 ligand, Poly(I:C) and Smac mimetic trigger necroptosis upon caspase inhibition in CCA cell lines. **a** RIPK3-expressing cells, KKU213 and HuCCT-1 were pretreated with 20 μM zVAD-fmk and Smac mimetic (5 nM KKU213 and 50 nM HuCCT-1) for 2 h. The cells were transfected with 2.5 μg/ml Poly(I:C) for 24 h and 48 h. TNF-α/zVAD-fmk/Smac mimetic (TSZ) were represented as a positive control. KKU213 (left) and HuCCT-1 (right) cells were pretreated with 10 μM RIPK3 inhibitor (GSK’872) (**b**) or 1 μM MLKL inhibitor (NSA) (**c**) for 2 h. At the same time, the cells were pretreated with zVAD-fmk and Smac mimetic (SZ). After that the cells were treated as in (**a**). **d** KKU213 and HuCCT-1 cells-expressing CRISPR control (CRISPR-V2) or CRISPR-RIPK3 (RIPK3) were treated as in (**a**) for 24 h. The representative knockout efficiency was shown on right. **e** KKU213 and HuCCT-1 cells-expressing shRNA control (shNT) or shRNAs targeting two different sequences of MLKL (shMLKL1, shMLKL2) were treated as in (**a**). The representative knockdown efficiency was shown on right. Cell death was determined by Annexin V and PI staining and flow cytometry. Data from three independent experiments was presented as mean ± S.D.; * *p* < 0.05, **p < 0.01, *** *p* < 0.001

TLR3-mediated cell death has been reported to induce not only apoptosis, but also necroptosis in cell lines with RIPK3 expression [45, 46]. Therefore, we hypothesized that the combination treatment in the presence of zVAD-fmk (Poly(I:C)/Smac/zVAD-fmk) could switch cell death mode to necroptosis in CCA cells-expressing RIPK3, since caspase inhibition has previously been reported to cause a switch from apoptosis to necroptosis [62]. To this end, we investigated key necroptotic proteins expression including RIPK1, RIPK3 and MLKL in a panel of CCA cell lines by Western blot analysis. RIPK1 and MLKL were similarly expressed in all CCA cell lines, whereas RIPK3 was only expressed in selected CCA cells including KKU213, RMCCA-1 and HuCCT-1 (Fig. S9). Therefore, the partial protection under the presence of zVAD-fmk in KKU213 which expressed RIPK3 might be due to a switch of cell death mode to necroptosis (Fig. 2a) but further investigations are required for clarification. To generalize our results in other CCA cells-expressing RIPK3, we therefore did a pilot study to screen for the sensitivity to Poly(I:C)/Smac/zVAD-fmk-induced cell death, both RMCCA-1 and HuCCT-1 exhibited sensitivity to Poly(I:C)/Smac/zVAD-fmk treatment, while MMNK1 was completely resistant (data not shown). Since the expression of TLR3 in HuCCT-1 was higher than RMCCA-1 and HuCCT-1 was more sensitive to Poly(I:C)/Smac/zVAD-fmk-induced cell death (Fig. 1e and Fig. S7D), we therefore selected this cell line for further experiments. Poly(I:C)/Smac/zVAD-fmk significantly induced cell death after 24 h and 48 h in both KKU213 and HuCCT-1 (Fig. 3a). We demonstrated that both GSK’872 and necrosulfonamide (NSA), RIPK3 and MLKL inhibitors, respectively reversed Poly(I:C)/Smac/zVAD-fmk-induced cell death in both KKU213 and HuCCT-1 cells, these effects were similar to TNF-α signaling serving as a positive control (Fig. 3b, c). In consistence with pharmacological inhibitors, CRISPR/cas9-mediated deletion of *RIPK3* and short hairpins (shRNAs) silencing of MLKL also significantly rescued Poly(I:C)/Smac/zVAD-fmk-induced cell death (Fig. 3d, e), but did not affect cell death in the absence of zVAD-fmk (data not shown). The knockout and knockdown efficiency was confirmed by Western blot analysis (Fig. 3d, e). Collectively, these results demonstrated that the combination treatment of TLR3 ligand, Poly(I:C) and Smac mimetic in the presence of zVAD-fmk triggers RIPK3- and MLKL-dependent necroptosis.

### TLR3 ligand, Poly(I:C) and Smac mimetic induce RIPK1 kinase-dependent both apoptosis and necroptosis in CCA cell lines

**Fig. 4:**
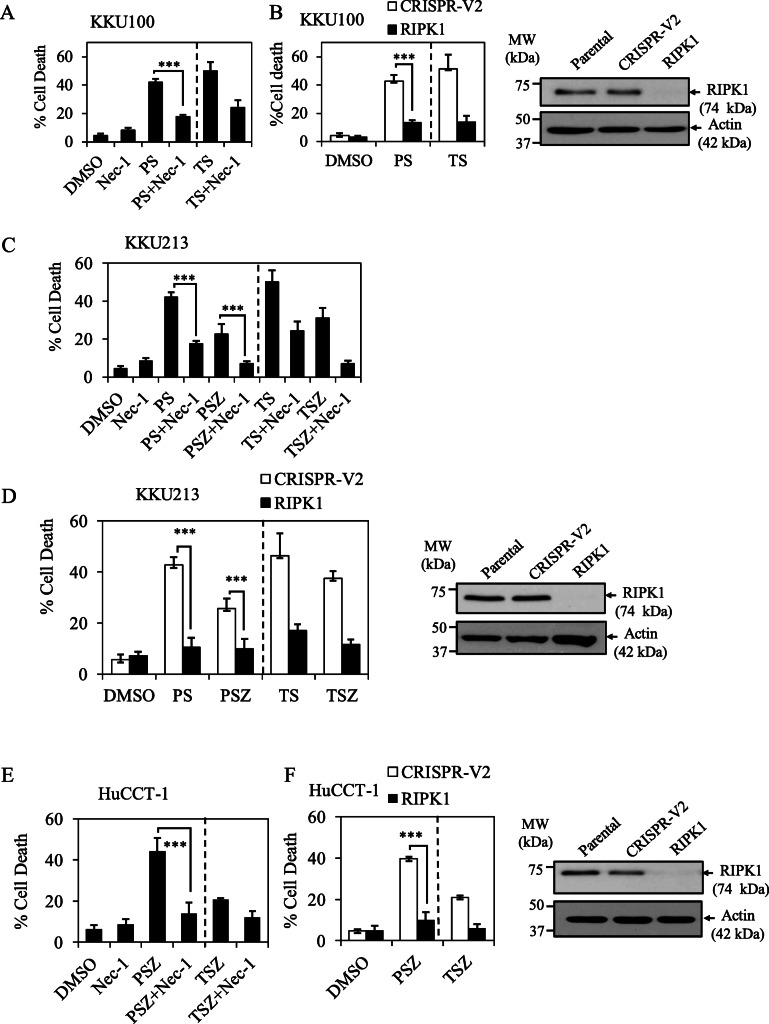
TLR3 ligand, Poly(I:C) and Smac mimetic induce RIPK1-dependent apoptosis and necroptosis in CCA cell lines. **a** KKU100 cells were pretreated with 60 μM RIPK1 inhibitor (Nec-1, necrostatin-1) and 50 nM Smac mimetic for 2 h followed by transfection with 2.5 μg/ml Poly(I:C) (PS) or treatment with 10 ng/ml TNF-α (TS) (positive control) for 24 h. **b** KKU100 cells-expressing CRISPR control (CRISPR-V2) or CRISPR-RIPK1 (RIPK1) were pretreated with 50 nM Smac mimetic for 2 h followed by treatment as in (**a**) for 24 h. **c** KKU213 cells was treated as in (**a**) except for Smac mimetic was used at 5 nM and zVAD-fmk was also included. **d** KKU213 cells-expressing CRISPR control (CRISPR-V2) or CRISPR-RIPK1 (RIPK1) were treated as in (**b**). **e** HuCCT-1 cells were pretreated with 50 nM Smac mimetic and zVAD-fmk for 2 h, followed by transfection with 2.5 μg/ml Poly(I:C) (PSZ) or treated with 10 ng/ml TNF-α (TSZ) (positive control) for 24 h. **f** HuCCT-1 cells-expressing CRISPR control (CRISPR-V2) or CRISPR-RIPK1 (RIPK1) were treated as in (**e**) for 24 h. The representative knockout efficiency in (**b**) KKU100, (**e**) KKU213, and (**f**) HuCCT-1 cells were shown on right. Cell death was determined by Annexin V and PI staining and flow cytometry. Data from three independent experiments was presented as mean ± S.D.; * *p* < 0.05, **p < 0.01, *** *p* < 0.001

RIPK1 was previously reported to act as a key mediator of TLR3-induced cell death by linking TLR3/TRIF to FADD/caspase-8 death complex [34]. We therefore hypothesized that RIPK1 could play a central mediator of TLR3 ligand, Poly(I:C) and Smac mimetic-induced both apoptosis (Poly(I:C)/Smac) and necroptosis (Poly(I:C)/Smac/zVAD-fmk) in CCA cell lines. RIPK1 inhibitor (Nec-1, necrostatin-1) significantly abolished Poly(I:C)/Smac-induced apoptosis in both KKU100 and KKU213 cell lines (Fig. 4a, c), but the protective effect was more pronounced in KKU100 and KKU213 when *RIPK1* was deleted by CRISPR/cas9 (Fig. 4b, d). In addition, both RIPK1 inhibitor (Nec-1) and genetic deletion of *RIPK1* by CRISPR/cas9 almost completely rescued Poly(I:C)/Smac/zVAD-fmk-induced necroptosis in CCA cell lines expressing-RIPK3 including KKU213 and HuCCT-1 (Fig. 4d, e, f). The knockout efficiency of RIPK1 was confirmed by Western blot analysis (Fig. 4b, d, f). These results all indicated that TLR3 ligand, Poly(I:C) and Smac mimetic induce both apoptosis and necroptosis in a RIPK1 kinase-dependent fashion in CCA cell lines.

### RIPK1 expression in primary CCA tissues and the expression status of RIPK1 and TLR3 on the survival of patients

**Fig. 5:**
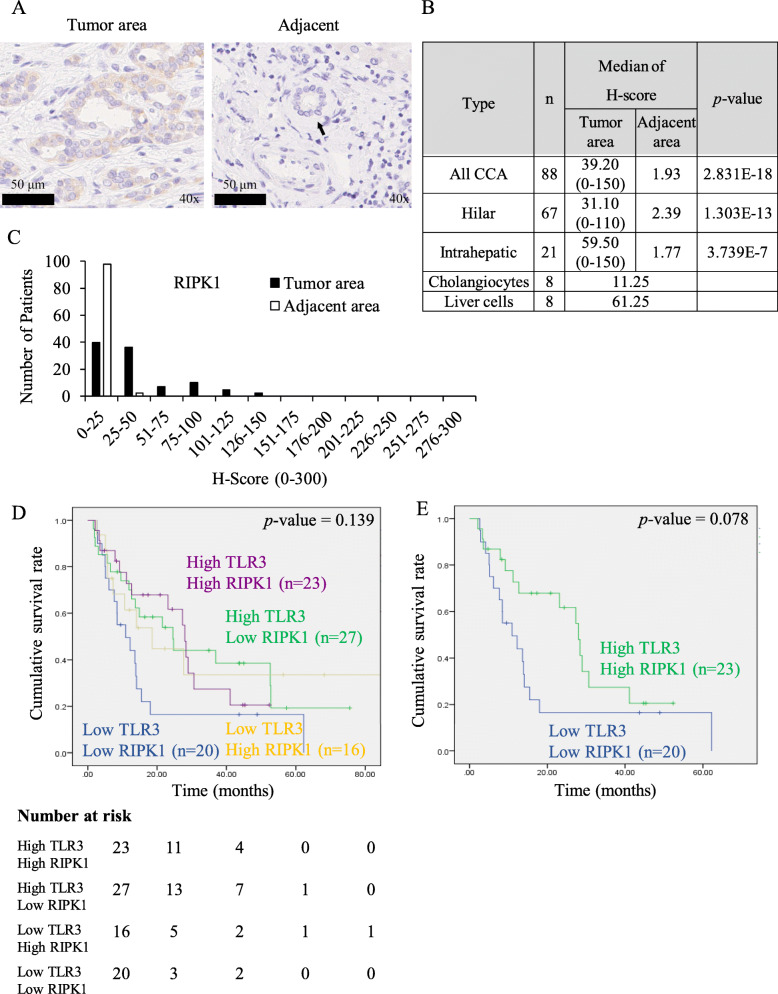
RIPK1 expression in primary CCA tissues and the expression status of RIPK1 and TLR3 on the survival of patients. **a** The representative cytoplasmic RIPK1 immunostaining in tumor area and adjacent normal tissues. Black arrowheads indicate bile ducts. **b** The median of H-score of RIPK1 staining in tumor area, adjacent normal tissues and cholangiocytes, liver cells in normal liver tissues. The difference between the median of H-score of tumor and adjacent tissues was calculated by one-way ANOVA and presented as *p*-value. **c** Distribution of RIPK1 expression according to H-score of tumor area and adjacent area. RIPK1 and TLR3 expression were classified into four groups according to low and high expression. Kaplan-Meier disease free survival curves stratified by (**d**) four groups of TLR3 and RIPK1 expression (Low Low, High High, Low High, High Low) and (**e**) two groups of TLR3 and RIPK1 expression (High High, Low Low)

RIPK1 represents a key mediator of TLR3 ligand and Smac mimetic induced both apoptosis and necroptosis in CCA cell lines, therefore investigation of RIPK1 in CCA patients became of great importance as an in vivo relevance for a potential therapeutic development. Therefore, in this study, RIPK1 was immunolocalized in 88 CCA patients (Fig. 5a, Fig. S10). The status of RIPK1 immunoreactivity in the cytoplasm of epithelial or parenchymal cells was significantly higher in CCA tissues than cholangiocytes adjacent to tumor tissues (*p* = 2.8312E-18) and cholangiocytes from normal liver tissues (Fig. 5b). The relative immunointensity of RIPK1 was low in CCA tissues but negative in adjacent tumor tissues (Fig. 5c). Kaplan-Meier survival analysis revealed no significant differences between high and low RIPK1 as well as TLR3 expression both disease-free survival (DFS) and overall survival (OS) rates (Fig. S11). Since RIPK1 and TLR3 might cooperatively influence the survival rate of the patients, we then attempted to combine RIPK1 and TLR3 status and tentatively classified into 4 subgroups. However, there were still no significant differences between low and high TLR3/RIPK1 in each subgroup (Fig. 5d). When compared between high TLR3/high RIPK1 and low TLR3/low RIPK1, there was a trend toward a longer DFS in patients with high TLR3 and high RIPK1 (*p* = 0.078) (Fig. 5e). The median of DFS in patients with high TLR3/high RIPK1 and low TLR3/low RIPK1 was 28.0 and 10.9 months, respectively. Altogether, these results indicated that RIPK1 and TLR3 are frequently expressed in CCA patients and patients with high TLR3 and high RIPK1 display a trend for a longer DFS that raise the possibility toward the therapeutic development targeting TLR3 signaling in combination with Smac mimetic.

### Loss of RIPK1 enhances TLR3 ligand, Poly(I:C)-induced CCA invasion and ERK activation

**Fig. 6:**
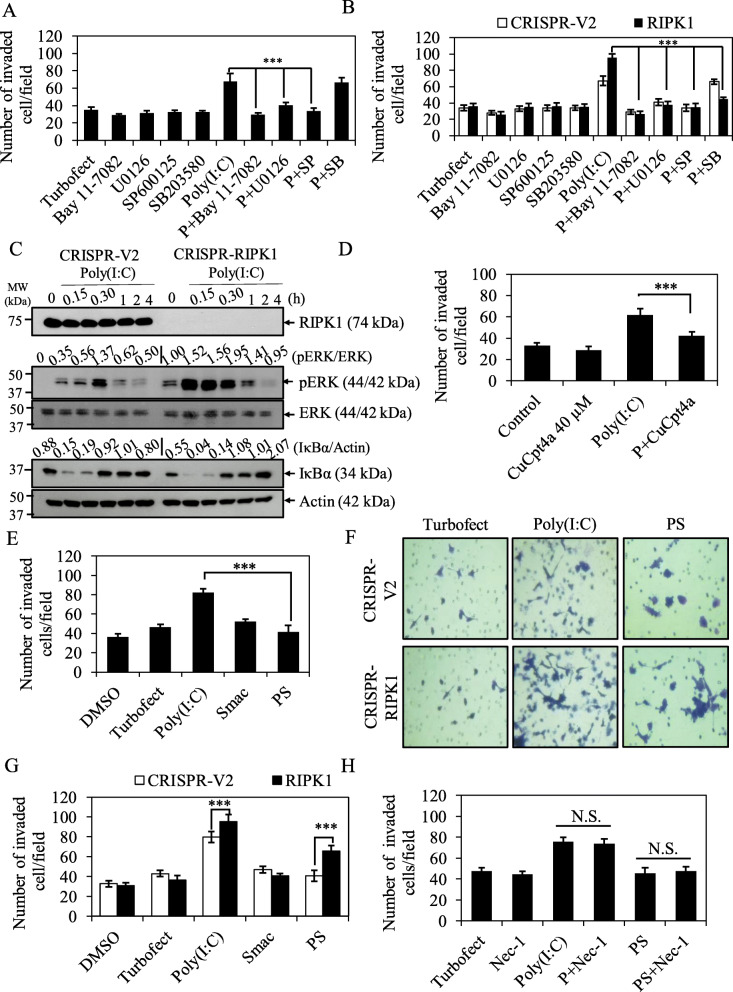
Smac mimetic reverses TLR3 ligand, Poly(I:C)-induced invasion in CCA cells. **a** KKU213 cells were pretreated with 10 μM Bay11–7082, 10 μM U0126, 20 μM SP600125 or 10 μM SB203580 for 30 min followed by transfection with TurboFect or 2.5 μg/ml Poly(I:C) for 12 h and then subjected to invasion assay. **b** CRISPR-V2 and CRISPR-RIPK1 KKU213 cells were treated as in (**a**) for 12 h and then subjected to invasion assay. **c** KKU213 cells were transfected with TurboFect or 2.5 μg/ml Poly(I:C) for indicated time points and cell lysates were collected, after that RIPK1, pERK, and IκBα were analyzed by Western blot analysis. Total ERK and β-actin were served as loading control. Data shown was a representative of two independent experiments. **d** KKU213 cells were pretreated with DMSO control or TLR3 inhibitor, CuCpt4a for 1 h followed by transfection with TurboFect or 2.5 μg/ml Poly(I:C) for 12 h and then subjected to invasion assay. **e** KKU213 cells were pretreated with 5 nM Smac mimetic for 2 h followed by transfection with 2.5 μg/ml Poly(I:C) for 12 h and then subjected to invasion assay. **f** Representative images of CRISPR-V2 and CRISPR-RIPK1 invaded cells stained with crystal violet. **g** Quantification of number of CRISPR-V2 and CRISPR-RIPK1 KKU213 invaded cells treated with 5 nM Smac mimetic, 2.5 μg/ml Poly(I:C) alone or the combination treatment for 12 h. **h** KKU213 cells were pretreated with RIPK1 inhibitor (Nec-1) with or without 5 nM Smac mimetic followed by transfection with 2.5 μg/ml Poly(I:C) for 12 h. Number of invaded cells were counted. Data from three independent experiments was presented as mean ± S.D.; * *p* < 0.05, **p < 0.01, *** *p* < 0.001, n.s. = not significant

We further analyzed the association of TLR3 or RIPK1 status with clinicopathological parameters by categorizing TLR3 and RIPK1 into high and low expression (median of H-score). As shown in Table 1, high TLR3 or low RIPK1 expression was significantly associated with perineural, vascular, and lymph node invasions in CCA patients. These results brought us to further explore TLR3 signaling in invasion and the contribution of RIPK1 to this process. In vitro Matrigel transwell invasion assay was set up in KKU213 with more invasive phenotype. KKU213 was treated with TLR3 ligand, Poly(I:C) and then invaded through Matrigel-coated transwell inserts for 12 h. As hypothesized, Poly(I:C)-treated KKU213 significantly exhibited higher number of invaded cells than transfection reagent (Turbofect) control groups (Fig. 6a). Poly(I:C)-induced invasion was significantly reduced by NF-κB inhibitor (Bay11–7082), pERK inhibitor (U0126) and pJNK inhibitor (SP600125), but not p38 inhibitor (SB203580) suggesting the involvement of NF-κB and MAPK signaling in Poly(I:C)-induced invasion (Fig. 6a). Since low RIPK1 expression was associated with more invasiveness in CCA patients, we therefore hypothesized that low or loss of RIPK1 expression might enhance Poly(I:C)-induced invasion. Of great interest, loss of RIPK1 in KKU213 cells significantly enhanced Poly(I:C)-induced invasion in NF-κB- and MAPK (pERK, pJNK and p38)-dependent manner (Fig. 6b). In addition, Poly(I:C)-induced ERK activation was more pronounced in RIPK1 knockout cells (Fig. 6c). Poly(I:C)-induced invasion was significantly inhibited by TLR3 inhibitor, CuCpt4a in both KKU213 and HuCCT-1 (Fig. 6d, Fig. S12) and TLR3 inhibitor also suppressed Poly(I:C)-induced TLR3 upregulation (Fig. S13) indicating that Poly(I:C)-induced invasion through TLR3 signaling. Altogether, these results suggested that loss of RIPK1 enhances TLR3 ligand, Poly(I:C)-induced CCA invasion and ERK activation.

### Smac mimetic reverses TLR3 ligand, Poly(I:C)-induced CCA invasion

Since Smac mimetic has been shown to reduce TRAIL-induced invasion in CCA cells [63], we therefore hypothesized that TLR3 ligand, Poly(I:C)-induced CCA invasion could be reversed by Smac mimetic. Interestingly, when Poly(I:C) was combined with Smac mimetic, the number of invaded cells was significantly lower when compared to Poly(I:C) treatment alone, whereas Smac mimetic did not affect the invaded cells (Fig. 6e). All treatment conditions at 12 h did not influence the cell proliferation or cell viability as evaluated by MTT assay (Fig. S14). The number of invaded cells was significantly higher in KKU213 RIPK1 knockout cells compared to control cells upon the combination treatment of Poly(I:C) and Smac mimetic, but still lower than the level in Poly(I:C)-treated RIPK1 knockout cells (Fig. 6f, g), suggesting that Smac mimetic inhibited Poly(I:C)-induced invasion, partly mediated by RIPK1. Similar findings were observed in another CCA cell line, HuCCT-1 (Fig. S15). In addition, RIPK1 inhibitor (Nec-1) harbored no effects on invasion either Poly(I:C) alone- or Poly(I:C) and Smac mimetic treatments (Fig. 6h), suggesting a RIPK1 kinase-independent. Taken together, our results suggested that Smac mimetic reverses TLR3 ligand, Poly(I:C)-induced CCA invasion, partly mediated through a RIPK1-dependent manner.

## Discussion

**Fig. 7:**
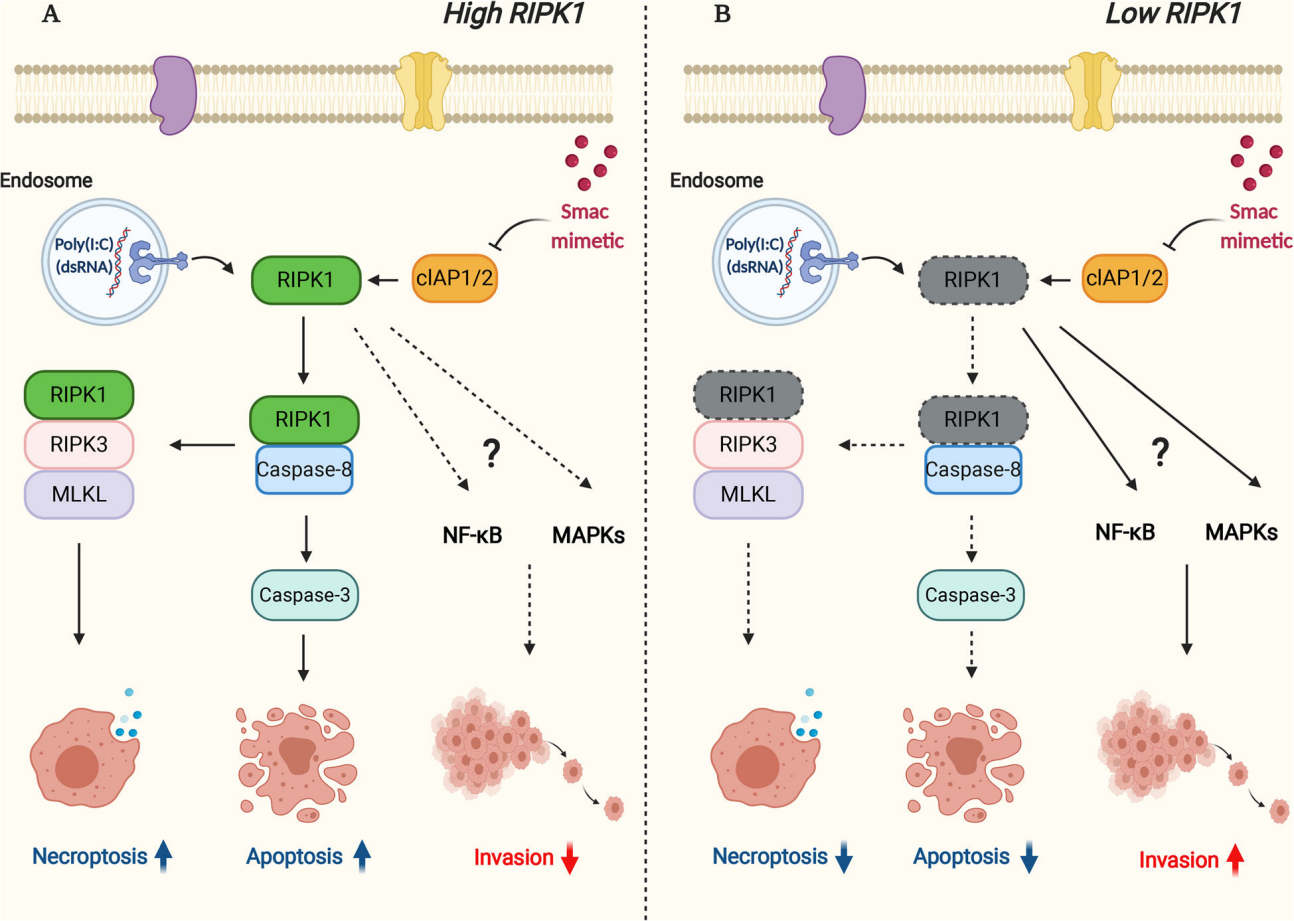
Schematic diagram of RIPK1 modulated TLR3 ligand, Poly(I:C)-induced cell death and invasion in CCA. **a** In CCA cells with RIPK1 and TLR3 expression, Smac mimetic and TLR3 ligand, Poly(I:C) induces apoptosis and necroptosis and Smac mimetic reverses Poly(I:C)-induced invasion. **b** In CCA cells with low RIPK1 expression, Smac mimetic and TLR3 ligand, Poly(I:C)-induced apoptosis and necroptosis are inhibited, while TLR3 ligand, Poly(I:C) enhances invasion. The reversion of Poly(I:C)-induced invasion by Smac mimetic is partly mediated by RIPK1. *Created with BioRender

Targeting TLR3 by TLR3 ligands has become an attractive therapeutic strategy in cancer immunotherapy but TLR3 activation in cancer cells could also trigger pro-tumorigenic effects. In this study, we demonstrated that low RIPK1 or high TLR3 status in tumor tissues was significantly associated with more invasiveness which was also confirmed by subsequent in vitro studies that the stimulation of TLR3 by TLR3 ligand, Poly(I:C) promoted CCA cell invasion in NF-κB and MAPK-dependent manner. The combined treatment of TLR3 ligand, Poly(I:C) and an IAP antagonist, Smac mimetic synergistically induced RIPK1 kinase-dependent apoptosis and necroptosis. Of particular interest, Smac mimetic also reversed TLR3 ligand-induced CCA cell invasion, which was partly mediated through RIPK1 (Fig. 7). Collectively, this is the very first study to demonstrate the interplay between RIPK1 and TLR3 signaling in CCA and therapeutic targeting TLR3 by TLR3 ligands in combination with Smac mimetic could bring a new therapeutic concept with more effective for CCA patients.

We first examined the expression of TLR3 in CCA patients. Consistent with results of the studies in other human malignancies [22, 33], results of our present study revealed that a large proportion of CCA patients were differentially expressed TLR3. Stimulation with TLR3 ligand, Poly(I:C) enhanced TLR3 expression in CCA cell lines, but not in nontumor cholangiocytes, suggesting that nontumor cholangiocytes might not be responsive to Poly(I:C) stimulation as TLR3 expression has previously been reported to be induced by Poly(I:C) [20, 64], probably through type I IFNs [65]. Therefore, the combination of Poly(I:C) with type I IFNs has been reported to enhance TLR3-induced cell death [20, 66]. In addition, TLR3 expression and activation were specific to tumor cells, which could also provide potential rationales for targeting TLR3 with more safe therapy. The status of TLR3 in cancer cells have been reported to predict favorable prognosis in neuroblastoma, HCC, NSCLC, and breast cancer [67–70]. In addition, in vivo anti-tumor effects of TLR3 ligand, Poly(I:C) are possibly mainly due to an induction of cell death upon direct stimulation of TLR3 by TLR3 ligand, Poly(I:C) [24] and also the recruitment of tumor-specific CD8+ T lymphocytes [71]. TLR3 ligand, Poly(I:C) stimulation has been reported to induce cell death on itself or combination with sensitizers in several cancers, but lack of evidence in CCA [18–31, 33–36]. Our results did demonstrate for the first time that Poly(I:C) itself did not induce CCA cell death but only in the presence of Smac mimetic, an IAP antagonist [72], the combination treatment significantly triggered apoptosis with high synergism. Surprisingly, this effect was by no means correlated with TLR3 levels in CCA cells, although TLR3 expression is proposed as a biomarker for the therapeutic efficacy of dsRNA in breast cancer patients [18]. However, it is entirely true that factors that might influence TLR3 ligand, Poly(I:C) responsiveness are not known at this juncture.

Notably, CCA cell lines that expressed key necroptotic proteins especially RIPK3 exhibited a switch to necroptosis. Necroptosis has been reported to enhance anti-tumor immunity in colon cancer and melanoma [41, 42] and RIPK3 expression status is proposed to influence the clinical outcome of TLR3-based cancer immunotherapy [45]. The loss of key necroptotic proteins in cancers has become a major hindrance for necroptosis-based therapy [73, 74] but results of our recent studies in CCA patients demonstrated that RIPK3 and MLKL were both expressed in a great majority of CCA patients, allowing for the possible development of necroptosis-based therapeutic approaches [75]. Collectively, these results provide a potential for development of a novel therapeutic approach targeting TLR3 by TLR3 ligands in combination with Smac mimetic that can trigger both apoptosis and necroptosis in a RIPK1-dependent manner. However, one of limitations in this study points to the fact that Poly(I:C), a synthetic analog of dsRNA can activate the signaling via both TLR3 and cytoplasmic dsRNA sensors such as melanoma differentiation-associated gene 5 (MDA5) and retinoic acid-inducible gene I (RIG-I). Although most studies have been demonstrated that Poly(I:C) induced apoptosis in a TLR3-dependent manner [34, 36, 61], our current study needs further characterization to support the specific role of TLR3-mediated apoptosis and necroptosis in CCA cells.

In our studies, we further demonstrated that low RIPK1 or high TLR3 in CCA patients was associated with more invasiveness. In vitro invasion assay further supports in vivo findings that stimulation of TLR3 by Poly(I:C) promoted CCA cell invasiveness through NF-κB and MAPK signaling. The activation of NF-κB and MAPK signaling after TLR4 and TLR3 stimulation has been demonstrated to promote invasion of lung cancer cells [76]. Similar to our findings, stimulation of TLR3 by Poly(I:C) induced migration and invasion in HNSCC, melanoma and lung cancer [29, 76–78]. Paradoxically, TLR3 ligand, Poly(I:C) have been reported to inhibit the migration in neuroblastoma and HCC [31, 32]. These results all indicated that TLR3 ligand, Poly(I:C)-induced migration and invasion is cell type-specific and context-dependent.

RIPK1 is well known to mediate both inflammation and cell death signaling [50], and has emerged as a critical regulator of cell fate determination in response to cellular stress [79]. Our results revealed that RIPK1 was required for TLR3 ligand, Poly(I:C) and Smac mimetic-induced both apoptosis and necroptosis in a kinase-dependent manner. The potential roles of RIPK1 in cell death regulation has been extensively reported but its roles in cancers have virtually remained debatable. Several previous studied in gallbladder carcinoma, melanoma, breast cancer, HCC, and CCA suggested that RIPK1 harbors pro-tumorigenic functions through different mechanisms including NF-κB, autophagy, and apoptosis activation [53, 80–85]. Therefore, targeting RIPK1 has been suggested as an attractive therapeutic target for those cancers. In contrast, recent study in HCC has revealed that low RIPK1 and TRAF2 expression was associated with unfavorable prognosis [86]. Mechanistically, loss of RIPK1 promotes HCC development in a kinase-independent manner through proteasomal degradation of TRAF2. Our results add more roles of RIPK1 in cancer, we showed that loss of RIPK1 expression enhanced Poly(I:C)-induced invasion in CCA cells. RIPK1 seems to negatively modulate Poly(I:C)-induced invasion in NF-κB and MAPK-dependent manner. In addition, loss of RIPK1 also markedly enhanced Poly(I:C)-induced ERK activation which may lead to more invasiveness, however the mechanism underlying this effect needs further investigation. In consistent with our study, shRNA silencing of RIPK1 in metastatic HNSCC enhances migration and low RIPK1 expression strongly correlates with metastatic phenotypes in HNSCC patients [54]. In contrast, silencing of RIPK1 expression inhibits invasion in gallbladder carcinoma, therefore RIPK1 might act as a double-edged sword in cancers [53].

TLR3 ligand-induced cancer cell death is a promising anti-cancer therapy, on another side we also demonstrated the pro-tumorigenic consequences of TLR3 ligand, Poly(I:C) that was discussed above. Of great interest, Smac mimetic reversed Poly(I:C)-induced CCA cell invasion to basal levels, adding more therapeutic benefits of Smac mimetic as a sensitizer of TLR3 ligand, Poly(I:C) treatment. Smac mimetic has been reported to reduce TRAIL-induced invasion and metastasis in CCA cells, partly explained by reducing TRAIL-induced NF-κB activation and thereby matrix metalloproteinase 7 (MMP7) expression [63]. Further studies are needed to investigate the underlying molecular mechanisms of how Smac mimetic reverses Poly(I:C)-induced invasion in CCA cells. As being targets of Smac mimetics, cIAPs might also contribute to promote Poly(I:C)-induced invasion in CCA cells, probably through NF-κB activation as previously reported for TNF-α signaling [51, 87, 88]. Our studies in CCA patients demonstrated that high expression of both TLR3 and RIPK1, although not significantly, but there was a trend toward a longer DFS in CCA patients (*p* = 0.078). Since CCA is associated with chronic inflammation, therefore the activation of TLR3 signaling in response to TLR3 ligands presented in CCA microenvironment might contribute to disease progression, however this pro-tumorigenic signaling might be negatively regulated in the presence of RIPK1. These results provide clinical significance to further support our studies that RIPK1 represents a key mediator in TLR3 ligand, Poly(I:C)-induced cell death and -inhibited invasion, therefore CCA patients with high TLR3 and high RIPK1 expression could be benefit for this novel treatment concept.

## Conclusion

We firstly demonstrated that the combination treatment of TLR3 ligand, Poly(I:C) and Smac mimetic induced both apoptosis and necroptosis in CCA cells but restricted to nontumor cholangiocytes. In addition, Smac mimetic also attenuated TLR3 ligand, Poly(I:C)-induced invasion. Therefore, therapy targeting TLR3 by TLR3 ligands in combination with Smac mimetic could provide a novel therapeutic concept with more effective for CCA patients. More importantly, this is the first study to demonstrate the dual roles of RIPK1 representing a key mediator in this treatment strategy by regulating both cell death and invasion of cancer cells. Finally, we proposed that the patients with high TLR3 and high RIPK1 could benefit greatly for a targeted and personalized therapy.

## Supplementary information

**Additional file 1: Fig. S1.** The representative immunohistochemical staining of TLR3 scored as 3 for strong staining, 2 for moderate staining, 1 for weak staining and 0 for no staining. **Fig. S2.** The sensitivity of CCA cell lines to Poly (I:C) transfection and direct treatment. **Fig. S3.** The expression of cIAP1, cIAP2, and cFLIP_L_. **Fig. S4.** The effect of Poly (I:C) and Smac mimetic combination treatment on cell death induction in CCA cell lines. **Fig. S5.** TLR3 ligand and Smac mimetic synergistically induce cell death in CCA cell lines. **Fig. S6.** Degradation of cIAP1 and cIAP2 by Smac mimetic, SM-164. **Fig. S7.** Upregulation of TLR3 by Poly (I:C). **Fig. S8.** Densitometry of representative Western blot in Fig. 2b. **Fig. S9.** The expression of key necroptotic proteins, RIPK1, RIPK3, and MLKL. **Fig. S10.** The representative immunohistochemical staining of RIPK1 scored as 3 for strong staining, 2 for moderate staining, 1 for weak staining and 0 for no staining. **Fig. S11.** Kaplan-Meier survival analysis of TLR3 or RIPK1. **Fig. S12.** TLR3 inhibits Poly (I:C)-induced invasion in HuCCT-1. **Fig. S13.** TLR3 inhibitor dose-dependently inhibits Poly (I:C)-induced TLR3 expression in KKU213 and HuCCT-1. **Fig. S14.** Cell viability/proliferation evaluated by MTT assay. **Fig. S15.** Smac mimetic reverse TLR3 ligand-induced invasion in HuCCT-1 cells.

## Data Availability

The datasets used and/or analyzed during the current study are available from the corresponding author on reasonable request.

## Abbreviations

TLR3: Toll-like receptor 3
TLRs: Toll-like receptors
CCA: Cholangiocarcinoma
RIPK1: Receptor-interacting protein kinase 1
RIPK3: Receptor-interacting protein kinase 3
MLKL: Mixed lineage kinase domain-like protein
cIAPs: Cellular inhibitor of apoptosis proteins
dsRNA: double-stranded RNA
IFNs: Interferons
TRIF: TIR-domain-containing adapter-inducing interferon-β
TRAF6: TNF receptor-associated factor 6
NF-κB: Nuclear factor kappaB
MAPK: Mitogen-activated protein kinase
IRF3: Interferon regulatory factor 3
Poly(I:C): Polyinosinic-polycytidylic acid
RCC: Renal cell carcinoma
HNSCC: Head and neck squamous cell carcinoma
HCC: Hepatocellular carcinoma
NSCLC: Non-small cell lung cancer
FADD: Fas-associated protein with death domain
ICD: Immunogenic cell death
RING: Really Interesting New Gene
c-FLIP: Cellular FLICE-like inhibitory protein
Nec-1: Necrostatin-1
NSA: Necrosulfonamide
DFS: Disease-free survival
OS: Overall survival
MDA5: Melanoma differentiation-associated gene 5
RIG-I: Retinoic acid-inducible gene I
