# Supplementary material for: Receptor-interacting protein kinase 1 is a key mediator in TLR3 ligand and Smac mimetic-induced cell death and suppresses TLR3 ligand-promoted invasion in cholangiocarcinoma
Source: Cell Commun Signal. 2020 Oct 9;18:161. doi: 10.1186/s12964-020-00661-3 (PMC7545934; doi:10.1186/s12964-020-00661-3)
Supplement: Supplementary file 2 — Additional file 1: Fig. S1. The representative immunohistochemical staining of TLR3 scored as 3 for strong staining, 2 for moderate staining, 1 for weak staining and 0 for no staining. Fig. S2. The sensitivity of CCA cell lines to Poly (I:C) transfection and direct treatment. Fig. S3. The expression of cIAP1, cIAP2, and cFLIPL. Fig. S4. The effect of Poly (I:C) and Smac mimetic combination treatment on cell death induction in CCA cell lines. Fig. S5. TLR3 ligand and Smac mimetic synergistically induce cell death in CCA cell lines. Fig. S6. Degradation of cIAP1 and cIAP2 by Smac mimetic, SM-164. Fig. S7. Upregulation of TLR3 by Poly (I:C). Fig. S8. Densitometry of representative Western blot in Fig. 2b. Fig. S9. The expression of key necroptotic proteins, RIPK1, RIPK3, and MLKL. Fig. S10. The representative immunohistochemical staining of RIPK1 scored as 3 for strong staining, 2 for moderate staining, 1 for weak staining and 0 for no staining. Fig. S11. Kaplan-Meier survival analysis of TLR3 or RIPK1. Fig. S12. TLR3 inhibits Poly (I:C)-induced invasion in HuCCT-1. Fig. S13. TLR3 inhibitor dose-dependently inhibits Poly (I:C)-induced TLR3 expression in KKU213 and HuCCT-1. Fig. S14. Cell viability/proliferation evaluated by MTT assay. Fig. S15. Smac mimetic reverse TLR3 ligand-induced invasion in HuCCT-1 cells. [file 12964_2020_661_MOESM2_ESM.pdf]

Supplementary Information

Receptor-interacting protein kinase 1 is a key mediator in TLR3 ligand and Smac mimetic-induced cell death and suppresses TLR3 ligand-promoted invasion in cholangiocarcinoma

Thanpisit Lomphithak[1], Swati Choksi[2], Apiwat Mutirangura[3], Rutaiwan Tohtong[4], Tewin Tencomnao[5], Hajime Usubuchi[6], Michiaki Unno[7], Hironobu Sasano[6], Siriporn Jitkaew[5]*

*Corresponding author:

Dr. Siriporn Jitkaew

Address: Age-Related Inflammation and Degeneration Research Unit, Department of Clinical Chemistry, Faculty of Allied Health Sciences, Chulalongkorn University, Bangkok, 10330, Thailand.

Email address: Siriporn.ji@chula.ac.th

Tel: (66) 022181081, Fax: (66) 022181082

Table of Content:

Supplementary Figures 1-15

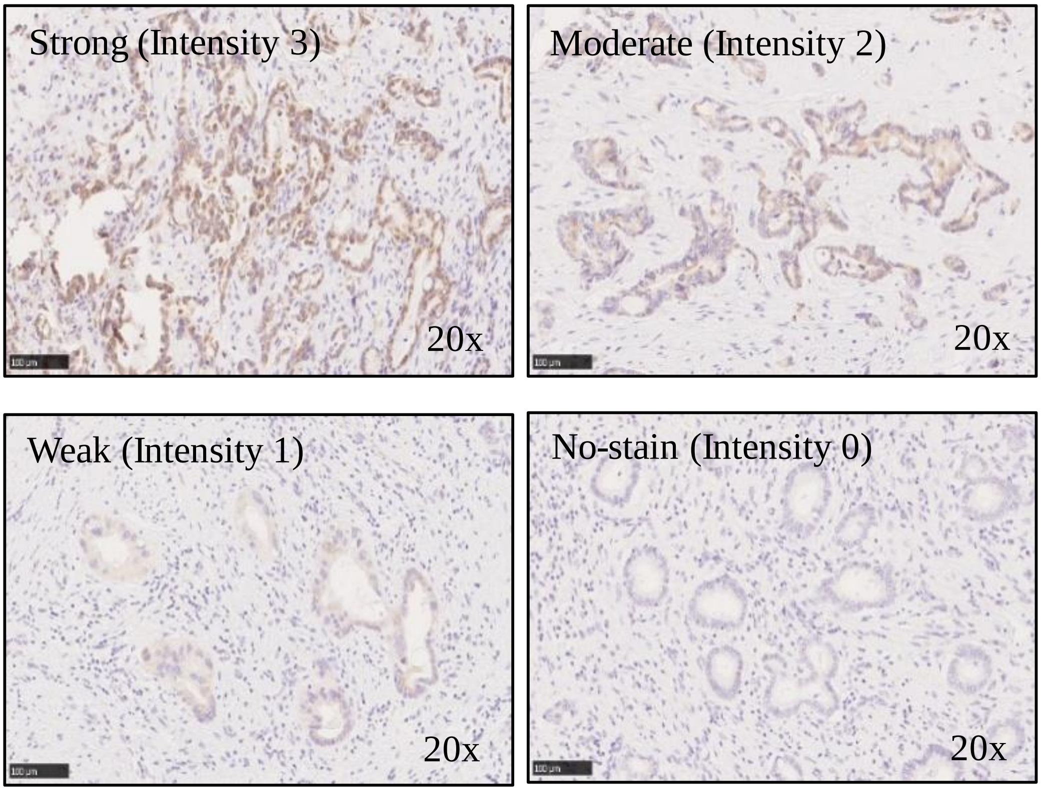

Fig. S1. The representative immunohistochemical staining of TLR3 scored as 3 for strong staining, 2 for moderate staining, 1 for weak staining and 0 for no staining.

A

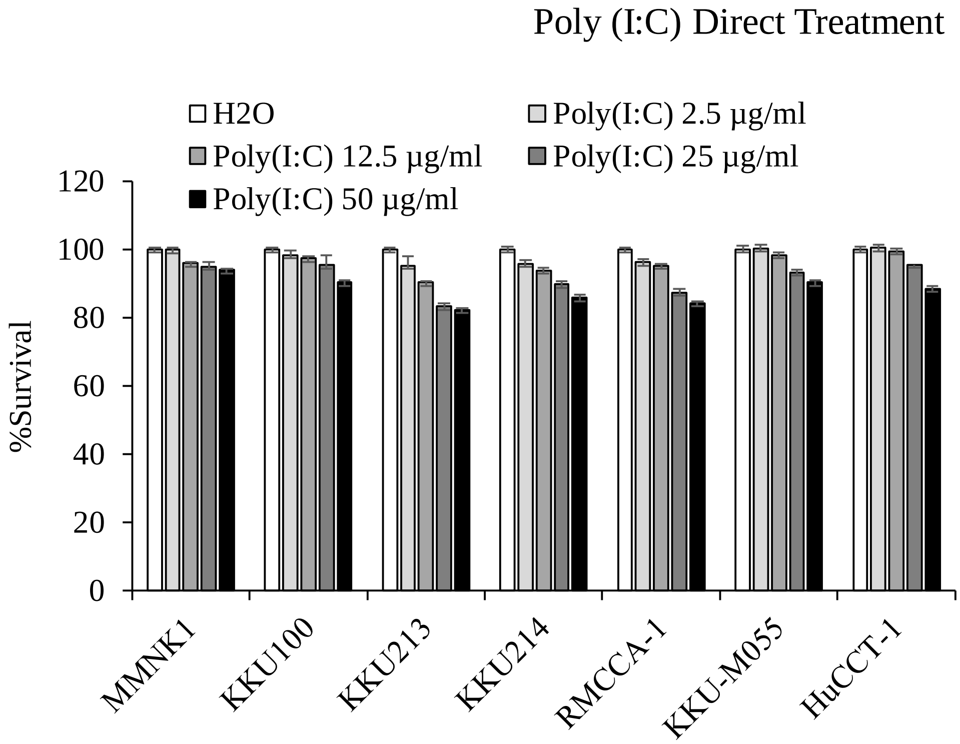

B

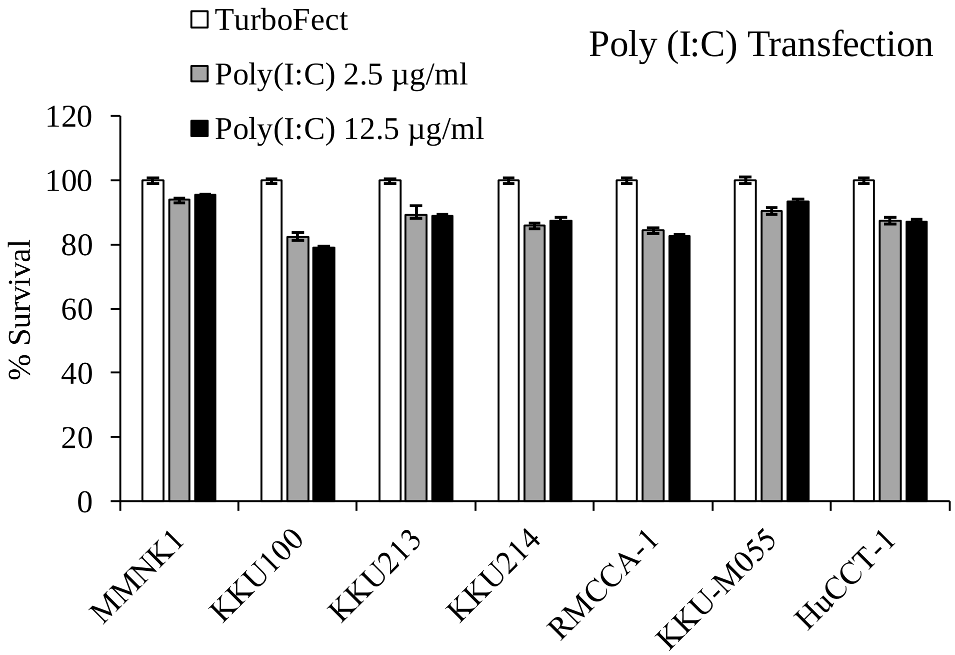

Fig. S2. The sensitivity of CCA cell lines to Poly(I:C) transfection and direct treatment. Six CCA cell lines and a nontumor cholangiocyte, MMNK1 were directly treated with different concentrations of Poly(I :C) at 2.5, 12.5, 25, and 50 µg/ml **(A)** or transfected with TurboFect or two different concentrations of Poly(I :C) at 2.5 µg/ml and 12.5 µg/ml **(B)** for 48 h. Cell viability was measured by MTT assay and % survival was calculated by normalizing Poly(I:C) treatment to vehicle control (H_2O) or TurboFect transfection reagent. Data from two independent experiments was presented as mean ± S.D.

A

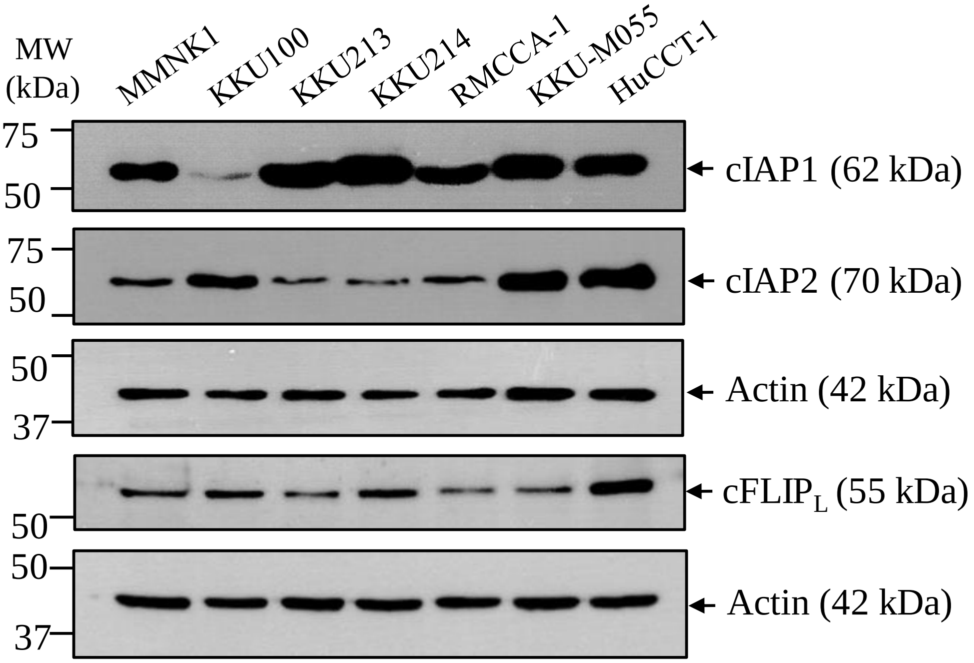

B

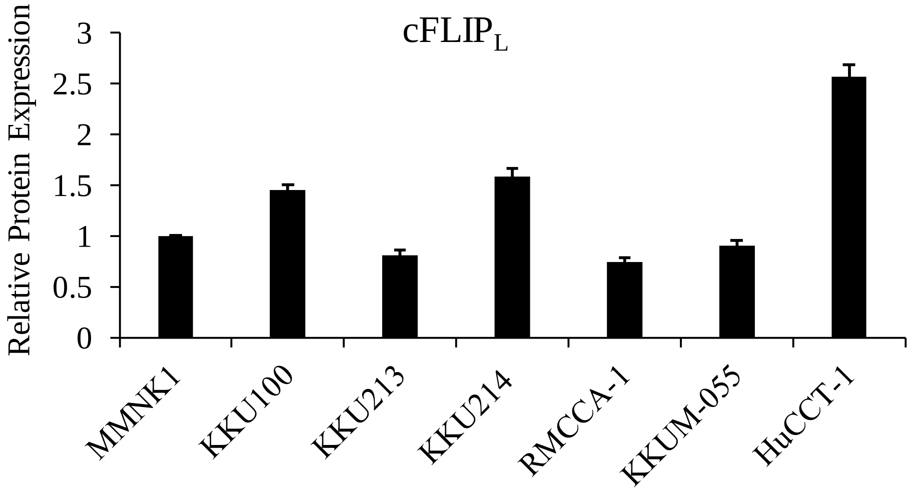

Fig. S3. The expression of cIAP1, cIAP2, and cFLIP$_L$. (**A**) Six CCA cells and a nontumor cholangiocyte, MMNK1 cell lysates were collected and subjected to Western blot analysis. β-actin was served as loading control. (**B**) cFLIP$_L$ was normalized to actin protein expression, and presented as fold increase relative to MMNK1 with its mean set to 1.

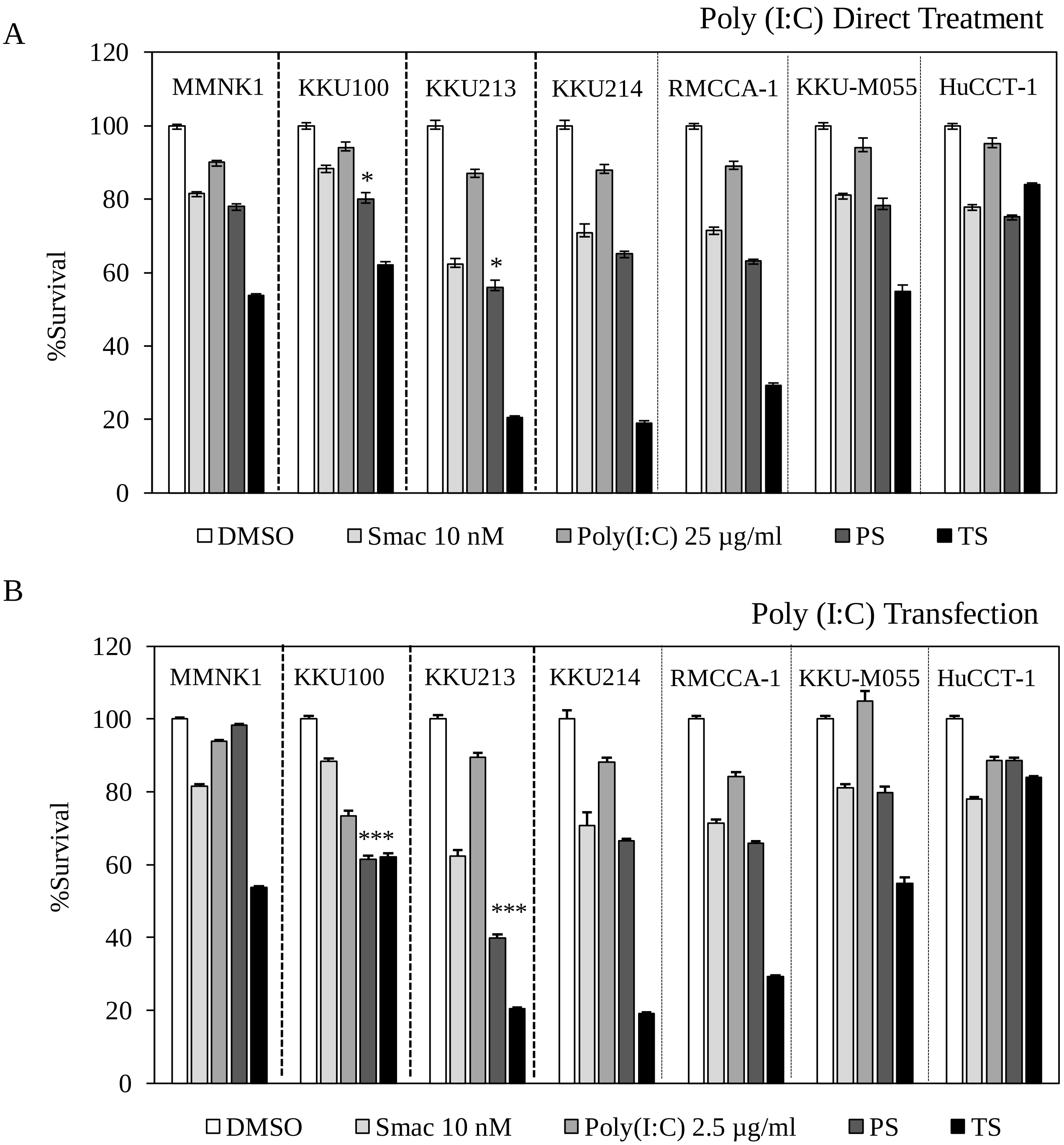

Fig. S4. The effect of Poly(I:C) and Smac mimetic combination treatment on cell death induction in CCA cell lines. Six different CCA cell lines and a nontumor cholangiocyte (MMNK1) were pretreated with 10 nM Smac mimetic (SM-164) for 2 h and were directly treated with 25 µg/ml Poly(I:C) (**A**) or were transfected with 2.5 µg/ml Poly(I:C) **(B)** for 24 h. TNF-α at 10 ng/ml and Smac mimetic at 10 nM (TS) were included as a positive control. Cell viability was determined by MTT assay. Data from two independent experiments was presented as mean ± S.D.; * $p < 0.05$, ** $p < 0.01$, *** $p < 0.001$.

A

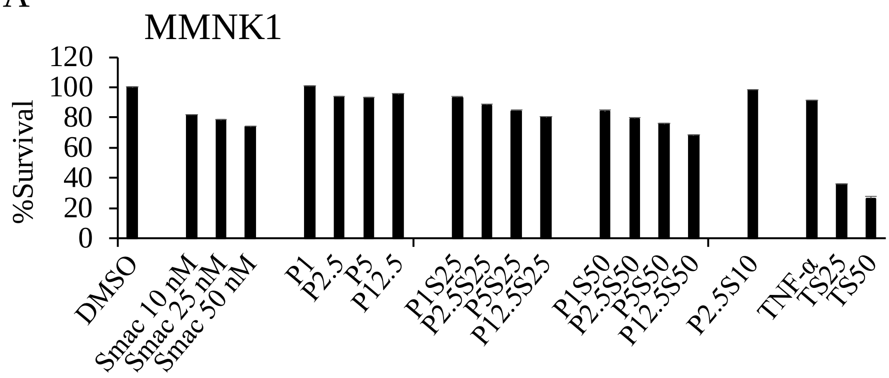

MNNK1		CI index		
Smac (nM)		10	25	50
Poly(I:C) (µg/ml)	1	ND	69.09	17.11
	2.5	16.51	52.14	3.13
	5	ND	9.16	1.18
	12.5	ND	3.12	0.99

B

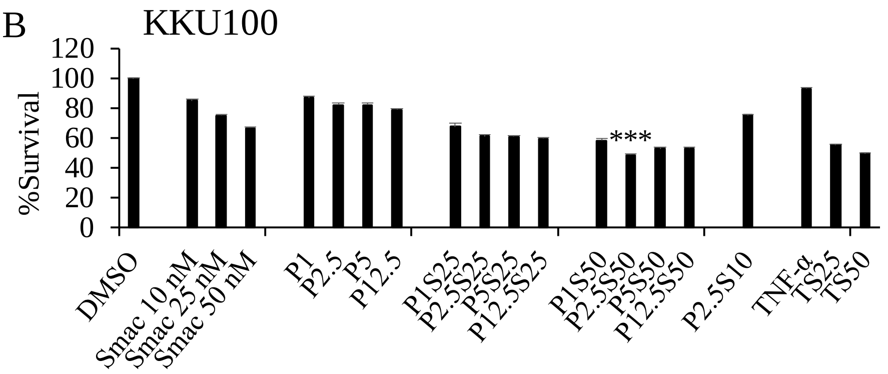

KKU100		CI index		
Smac (nM)		10	25	50
Poly(I:C) (µg/ml)	1	ND	0.58	0.61
	2.5	0.53	0.38	**0.34**
	5	ND	0.39	0.45
	12.5	ND	0.41	0.47

C

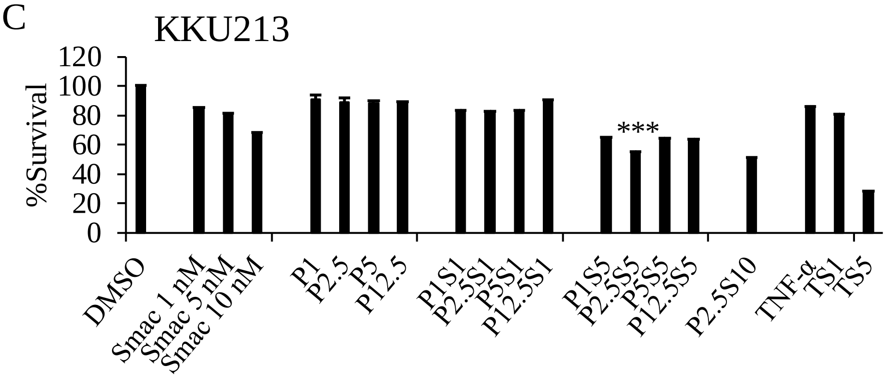

KKU213		CI index		
Smac (nM)		1	5	10
Poly(I:C) (µg/ml)	1	0.64	0.20	ND
	2.5	0.56	**0.07**	0.49
	5	0.65	0.19	ND
	12.5	1.14	0.17	ND

Fig. S5. TLR3 ligand and Smac mimetic synergistically induce cell death in CCA cell lines. (**A**) MMNK1, (**B**) KKU100, and (**C**) KKU213 were pretreated with indicated concentration of Smac mimetic and were transfected with different concentrations of Poly(I:C) (1, 2.5, 5, and 12.5 µg/ml) for 24 h. Cell viability was determined by MTT assay. The Chou-Talalay method was used to calculate combination index (CI) where C > 1 antagonism, CI = 0.9-1 additive, and CI < 0.9 synergism. Data from three independent experiments was presented as mean ± S.D.; * $p < 0.05$, ** $p < 0.01$, *** $p < 0.001$.

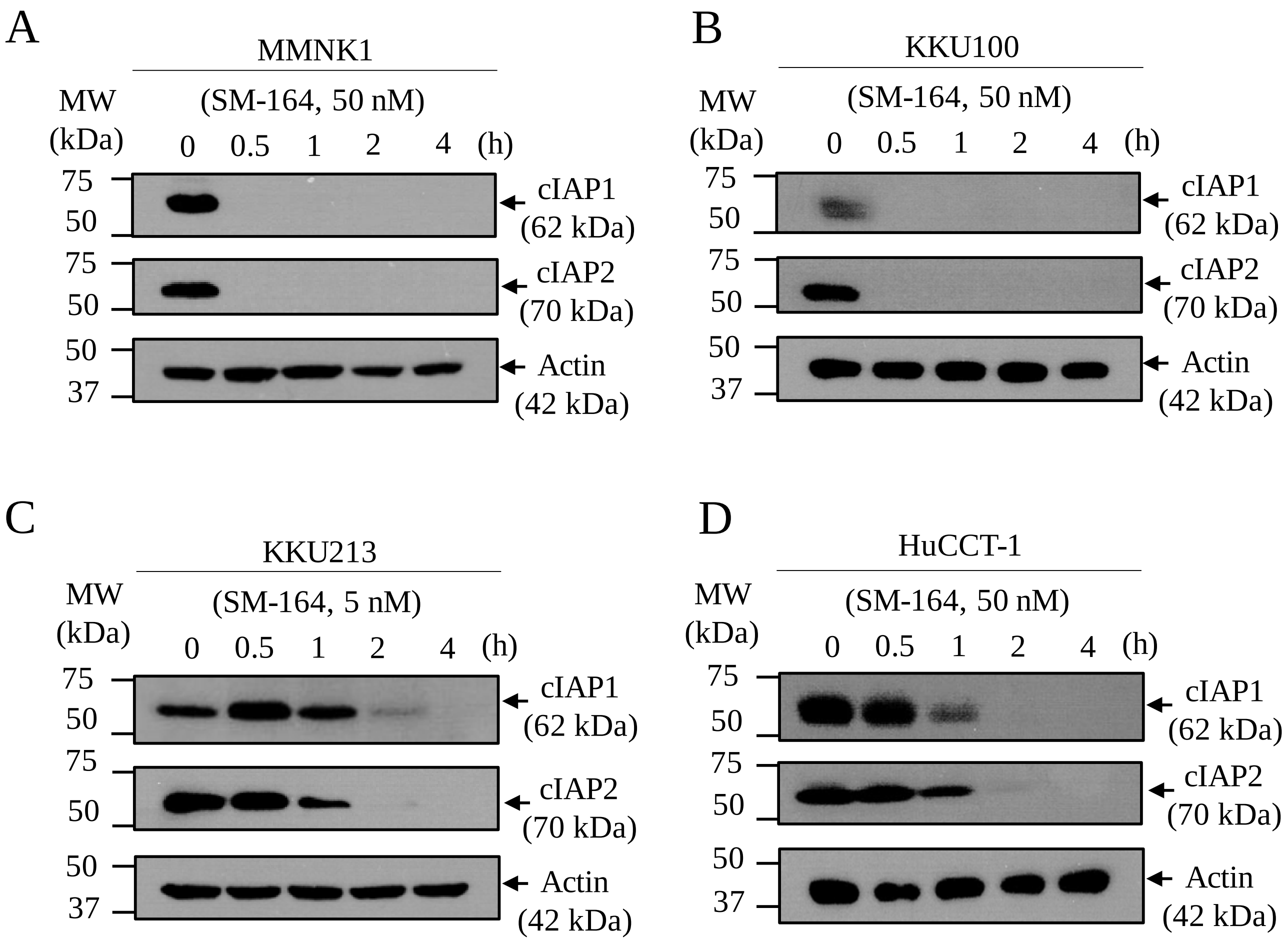

Fig. S6. Degradation of cIAP1 and cIAP2 by Smac mimetic, SM-164. (**A**) MMNK1, (**B**) KKU100, (**C**) KKU213, and (**D**) HuCCT-1 cells were treated with Smac mimetic at indicated concentrations and time points. The expression of cIAP1 and cIAP2 was determined by Western blot analysis. β-actin was served as loading control.

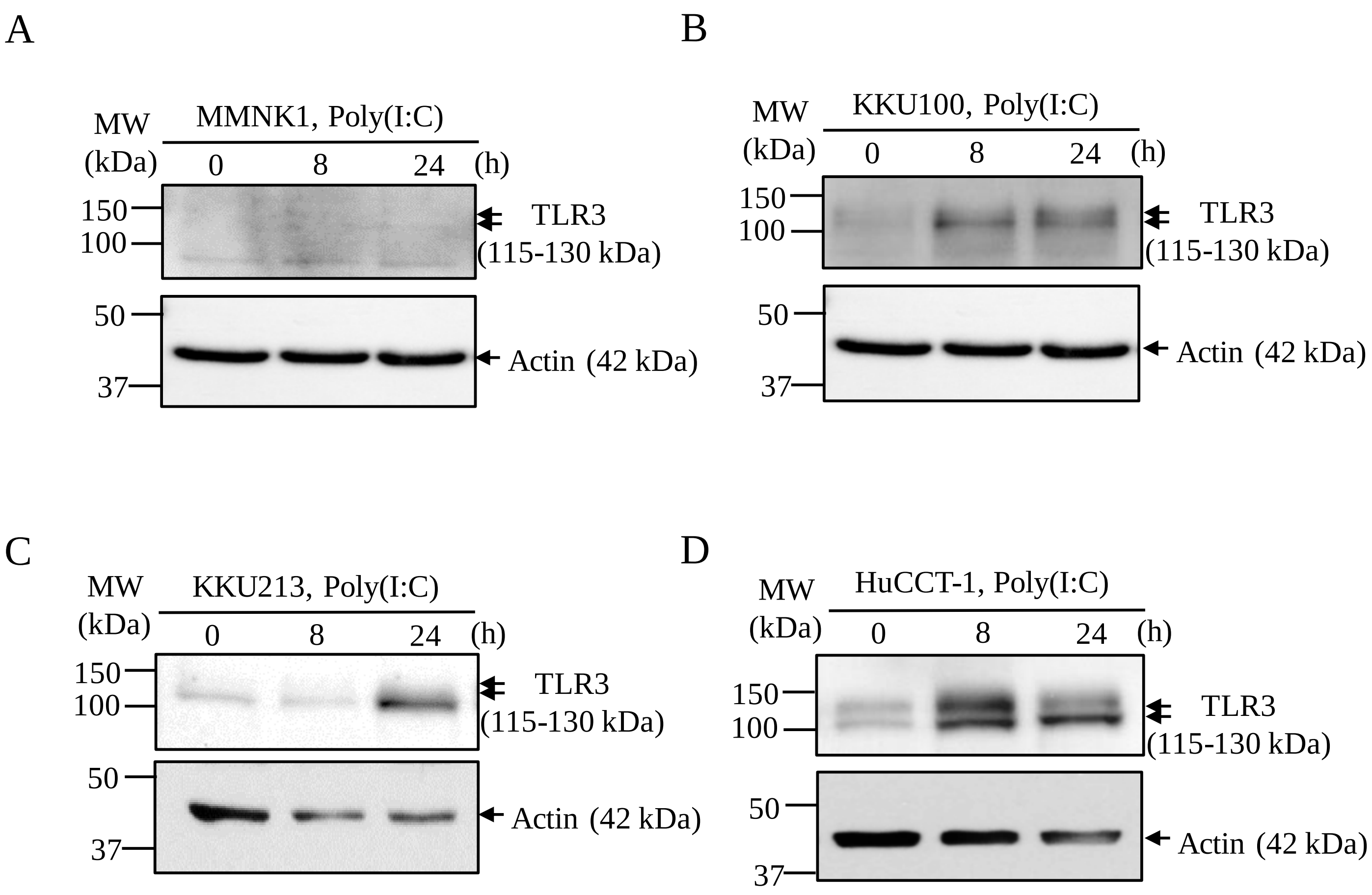

Fig. S7. Upregulation of TLR3 by Poly(I:C). (**A**) MMNK1, (**B**) KKU100, (**C**) KKU213, and (**D**) HuCCT-1 cells were transfected with 2.5 µg/ml Poly(I:C) at indicated time points. The expression of TLR3 was determined by Western blot analysis. β-actin was served as loading control.

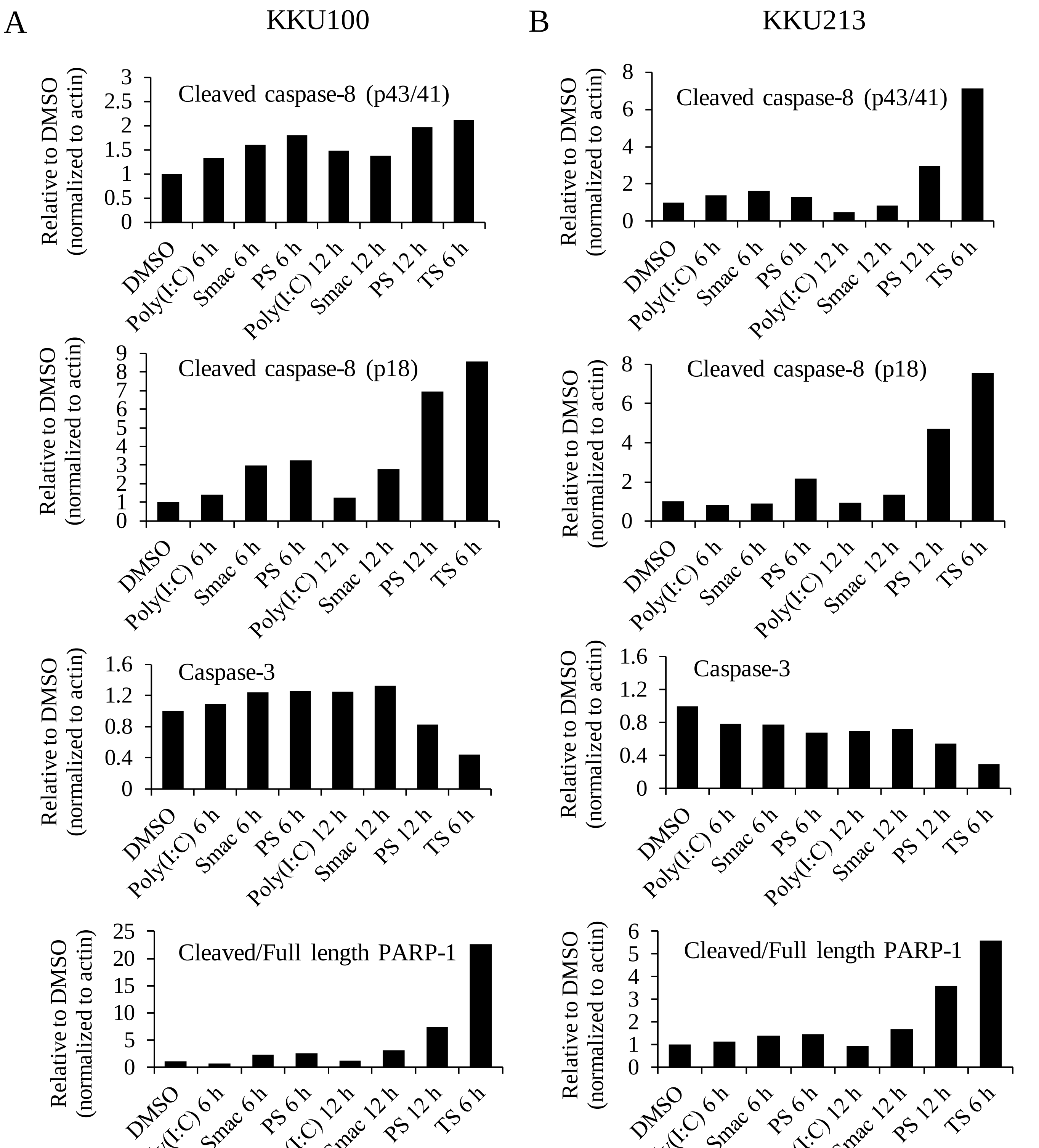

Fig. S8. Densitometry of representative Western blot in Figure 2b. (A) KKU100 and (**B**) KKU213, Western blot bands of cleaved caspase-8 (p43/p41), cleaved caspase-8 (p18), caspase-3, cleaved/full length PARP1 were analyzed by densitometry. Intensity of each band was normalized to actin and presented as fold changes relative to DMSO with its mean set to 1.

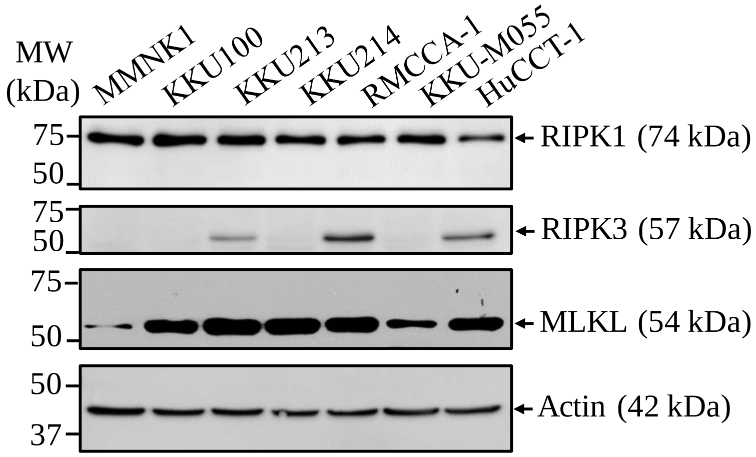

Fig. S9. The expression of key necroptotic proteins, RIPK1, RIPK3, and MLKL. Six CCA cell lines and a nontumor cholangiocyte, MMNK1 cell lysates were collected and subjected to Western blot analysis. β-actin was served as loading control.

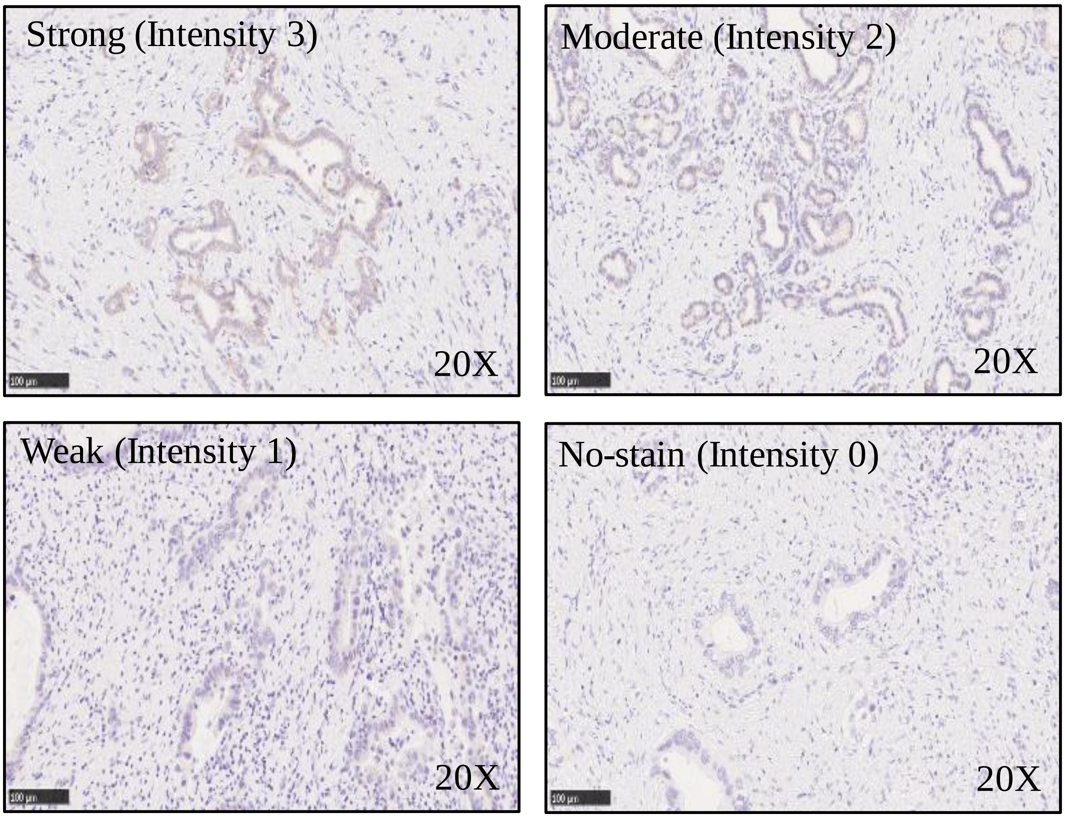

Fig. S10. The representative immunohistochemical staining of RIPK1 scored as 3 for strong staining, 2 for moderate staining, 1 for weak staining and 0 for no staining.

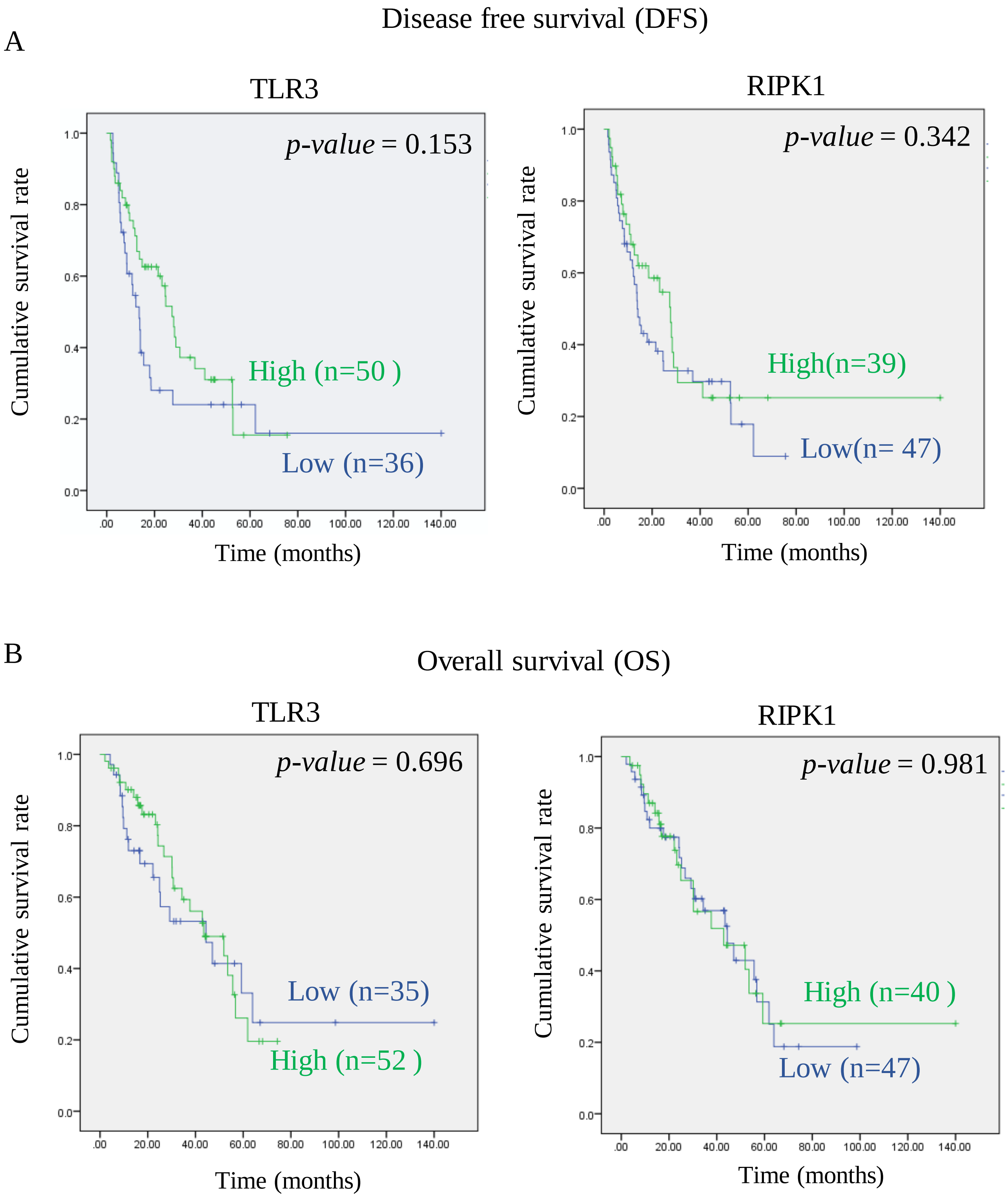

Fig. S11. Kaplan-Meier survival analysis of TLR3 or RIPK1. (**A**) Disease free survival (**B**) Overall survival

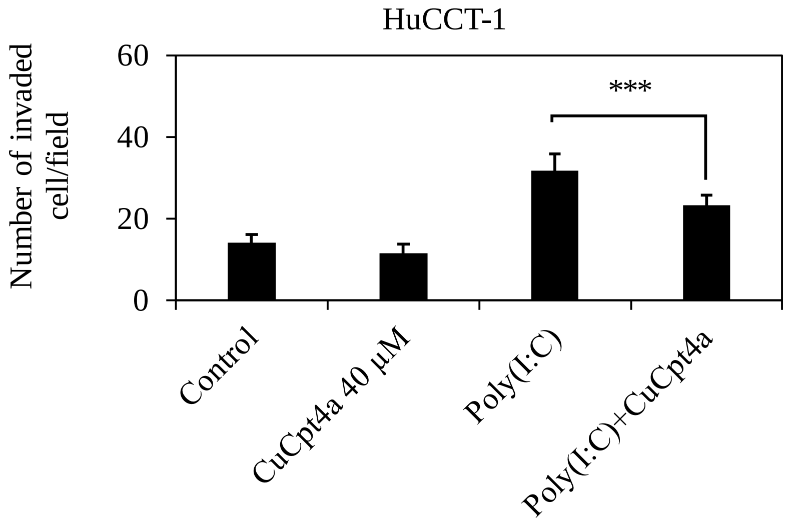

Fig. S12. TLR3 inhibitor inhibits Poly(I:C)-induced invasion in HuCCT-1. HuCCT-1 cells were pre-treated with DMSO control or 40 µM CuCpt4a for 1 h followed by transfection with TurboFect or 2.5 µg/ml Poly(I:C) and then subjected to invasion assays for 12 h. Number of invaded cells were counted. Data from two independent experiments was presented as mean ± S.D. ; * p < 0.05, **p < 0.01, *** p < 0.001.

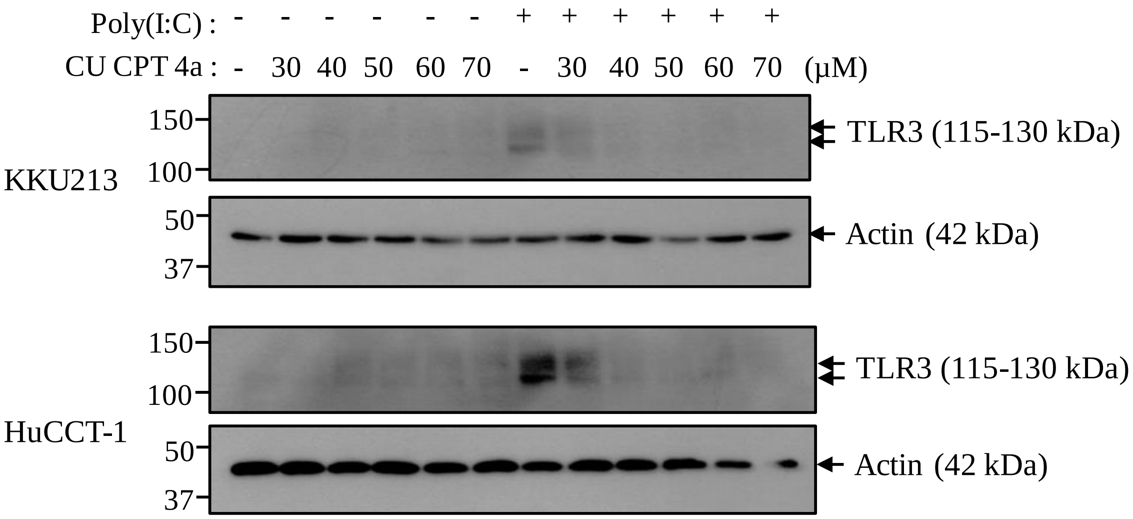

Fig. S13. TLR3 inhibitor dose-dependently inhibits Poly(I:C)-induced TLR3 expression in KKU213 and HuCCT-1. KKU213 and HuCCT-1 cells were pre-treated with DMSO control or indicated concentration of CuCpt4a for 1 h followed by transfection with TurboFect or 2.5 µg/ml Poly(I:C) for 24 h. TLR3 expression was determined by Western blot analysis. Actin was used as a loading control.

A

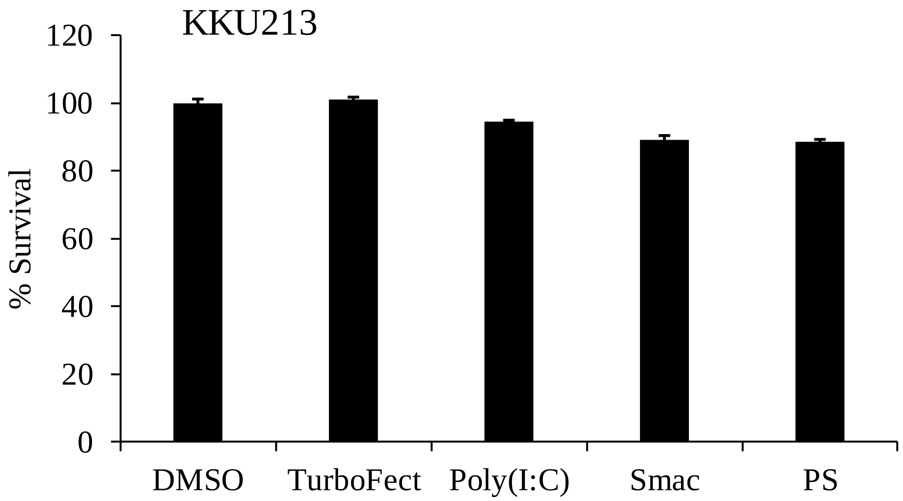

B

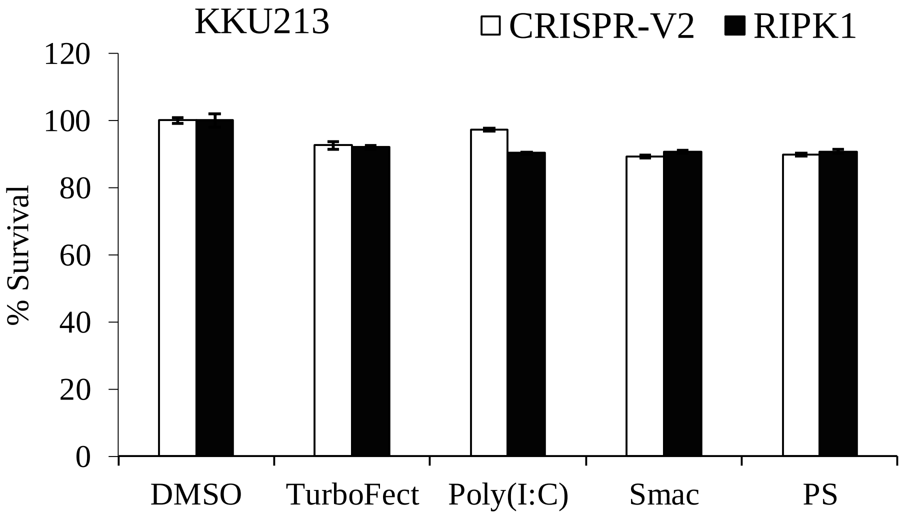

Fig. S14. Cell viability/proliferation evaluated by MTT assay. (A) KKU213 parental cells and KKU213 cells-expressing CRISPR control (CRISPR-V2) or CRISPR-RIPK1 (RIPK1) were pretreated with DMSO or 5 nM Smac mimetic (Smac) followed by transfected with TurboFect or 2.5 µg/mL Poly(I:C) for 12 h. Cell viability was measured by MTT assay and % survival was calculated by normalizing treatment to control (DMSO). Data from two independent experiments was presented as mean ± S.D.

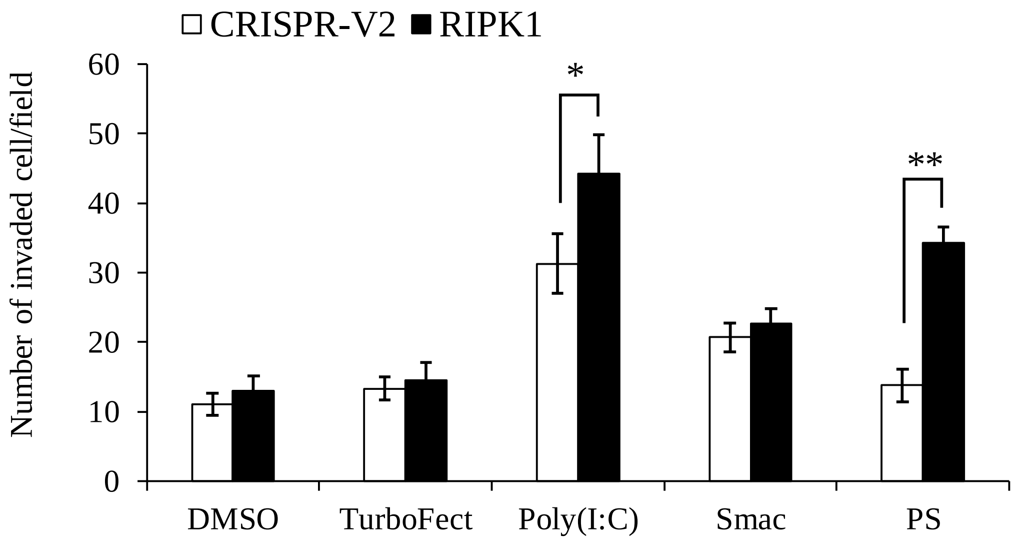

Fig. S15. Smac mimetic reverses TLR3 ligand-induced invasion in HuCCT-1 cells. HuCCT-1 cells-expressing CRISPR-V2 and CRISPR-RIPK1 were pre-treated with DMSO control or 50 nM Smac mimetic followed by transfection with TurboFect or 2.5 µg/ml Poly(I:C) and then subjected to invasion assays for 12 h. Number of invaded cells were counted. Data from two independent experiments was presented as mean ± S.D.; * $p < 0.05$, ** $p < 0.01$, *** $p < 0.001$.
